# A fluorogenic ROS-triggered hydrogen sulfide donor for alleviating cerebral ischemia-reperfusion injury

**DOI:** 10.7150/thno.100930

**Published:** 2024-11-04

**Authors:** Huangjie Lu, Huiying Zeng, Wenlong Wei, Yuying Chen, Ziqiang Zhou, Xuyang Ning, Ping Hu

**Affiliations:** 1 Department of Burns & Plastic Surgery, Guangzhou Red Cross Hospital, Faculty of Medical Science, Jinan University, Guangzhou 510006, China; 2 State Key Laboratory of Bioactive Molecules and Druggability Assessment, Jinan University, Guangzhou 510006, China.; 3 College of Pharmacy, Jinan University, Guangzhou 510006, China.

**Keywords:** Hydrogen sulfide donor, Reactive oxygen species, Molecular imaging, Cerebral ischemia-reperfusion injury, Theranostic agent

## Abstract

**Rationale:** Cerebral ischemia-reperfusion injury is a severe neurovascular disease that urgently requires effective therapeutic interventions. Recently, hydrogen sulfide (H_2_S) has garnered significant attention as a potential treatment for stroke; however, the precise and targeted delivery of H_2_S remains a considerable challenge for its clinical application.

**Methods:** We have developed HSDF-NH_2_, a novel H_2_S donor characterized by high selectivity, self-reporting capabilities, and the ability to penetrate the blood-brain barrier (BBB).

**Results:** HSDF-NH_2_ effectively scavenges reactive oxygen species (ROS) while generating H_2_S, with emitted fluorescence facilitating the visualization and quantification of H_2_S release. This compound has demonstrated protective effects against cerebral ischemia-reperfusion (I/R) injury and contributes to the reconstruction of brain structure and function in a rat stroke model (tMCAO/R).

**Conclusion:** As a ROS-responsive, self-reporting, and fluorescent H_2_S donor, HSDF-NH_2_ holds considerable promise for the treatment of ischemic diseases beyond stroke.

## Introduction

Stroke is an acute cerebrovascular disease and the second leading cause of death worldwide 1. It is characterized by high mortality, morbidity, disability and recurrence 2. Notably, ischemic stroke constitutes approximately 80% of all stroke cases. Ischemic stroke triggers a series of biochemical cascades that result in metabolic abnormalities in brain tissue, leading to a variety of pathological changes including neuronal damage, neuroinflammation and oxidative stress 3. Currently, the main strategies for treating ischemic stroke include thrombolysis (reperfusion) and neuroprotection 4. Among them, reperfusion therapy through the use of recombinant tissue plasminogen activator (rt-PA) 5 or mechanical means remains an urgent option for the treatment of acute ischemic stroke, but its application is limited due to the narrow therapeutic window and serious risks such as intracerebral hemorrhage 6. In addition, oxidative stress will suddenly occur after thrombolysis and large amounts of reactive oxygen species (ROS) will be produced, leading to various oxidative damages, including the production of proinflammatory cytokines, inflammatory infiltration, glial cell activation, etc., thereby aggravating secondary cerebral ischemia-reperfusion (I/R) injury 7-9. Thus, there is an urgent need to develop new, effective treatment strategies for ischemic stroke.

Neurons in the penumbra of the stroke-affected brain retain salvageable bio-functional activity, making the use of neuroprotective drugs beneficial for reducing oxidative stress and neuroimmune inflammation to rescue damaged cells 10. Unfortunately, many neuroprotective agents face significant limitations such as poor solubility, short half-lives, and inadequate penetration of the BBB, hindering their ability to accumulate and achieve effective therapeutic outcomes at the lesion site 11, 12. Hydrogen sulfide (H₂S) acts as a gaseous signaling molecule, exerting significant biological effects in various physiological and pathological processes 13. It also holds substantial therapeutic potential. Research studies in recent years have confirmed the positive effect of suitable concentrations of H_2_S in the treatment of ischemic stroke 14. H_2_S can mitigate ischemic brain injury by increasing superoxide dismutase (SOD) activity and glutathione peroxidase (GSH-PX) expression, lowering malondialdehyde (MDA) levels, and selectively eliminating excessive ROS 15-17. H_2_S can ameliorate brain tissue oedema by reducing the production of TNF-α and some other inflammatory mediators 18, as well as attenuating local inflammatory responses by inhibiting NF-κB nuclear translocation 19. In addition, also H_2_S can play a crucial protective role against cerebral ischemia/reperfusion injury by attenuating cerebrovascular endothelial cell injury, suppressing apoptosis inhibition, and mitochondrial calcium overloading 20. However, this bioactive gas is characterized by rapid evaporation and high reactivity, and achieving precise dose and *in vivo* distribution tracking of H_2_S is very difficult. These challenges seriously hinder the clinical translation of H_2_S-based therapies and the mechanistic understanding of their biological effects. Therefore, it is crucial to develop novel hydrogen sulfide donors to overcome the aforementioned application issues.

To investigate the physiological and pathophysiological properties of H_2_S, numerous H_2_S donors have been developed and reported. Inorganic H_2_S donors (e.g. NaHS and Na_2_S) are the most commonly used hydrolysis-mediated donors for basic research, but hydrolysis results in a rapid release of H_2_S and H_2_S escape is observed and does not realistically mimic the biological effects of endogenous H_2_S 21. Lawesson's Reagent (LR) was originally developed as a sulfurizing agent capable of releasing H_2_S more slowly than sulfide salts in aqueous solution, but it was gradually phased out due to its low aqueous solubility, limited release kinetic properties and unclear release mechanism. GYY4137, as an improved derivative of water-soluble Lawson's reagent, not only releases low concentrations of free H_2_S molecules in a sustained manner, but also has good biocompatibility. However, it is typically synthesized and marketed as a dichloromethane complex, and metabolizes another signaling molecule (i.e., CO) *in vivo*, affecting the biological effects of H_2_S 22. Recent studies have shown that the applications of H_2_S donors including NaHS, Na_2_S, GYY4137, etc. have spanned a wide range of fields such as cardiovascular, neurological, anti-inflammatory, and anticancer fields, and have great potential for a wide range of medical and biological studies such as nanomedicine, medical research, clinical trials, and drug design and optimization 23. In recent years, researchers have often introduced H_2_S releasing fractions into the parent body in order to improve the pharmacological/therapeutic properties of clinical drugs 24-26. H_2_S release from these donors can be achieved by a variety of mechanisms, including photoactivation 27-31, enzymolysis 32-34, free thiol (cysteine and reduced glutathione) activation 35-44, ROS response 45-47, and other response mechanisms. Notably, ROS-responsive H_2_S donors have garnered significant attention in recent years. Pluth *et al.* reported in 2016 that caged carbonyl sulfide (COS) binds to a ROS-responsive aryl borate as a COS/H_2_S donor, which specifically responds to and depletes cellular ROS and then releases an equivalent amount of H_2_S 45.

**Scheme 1:**
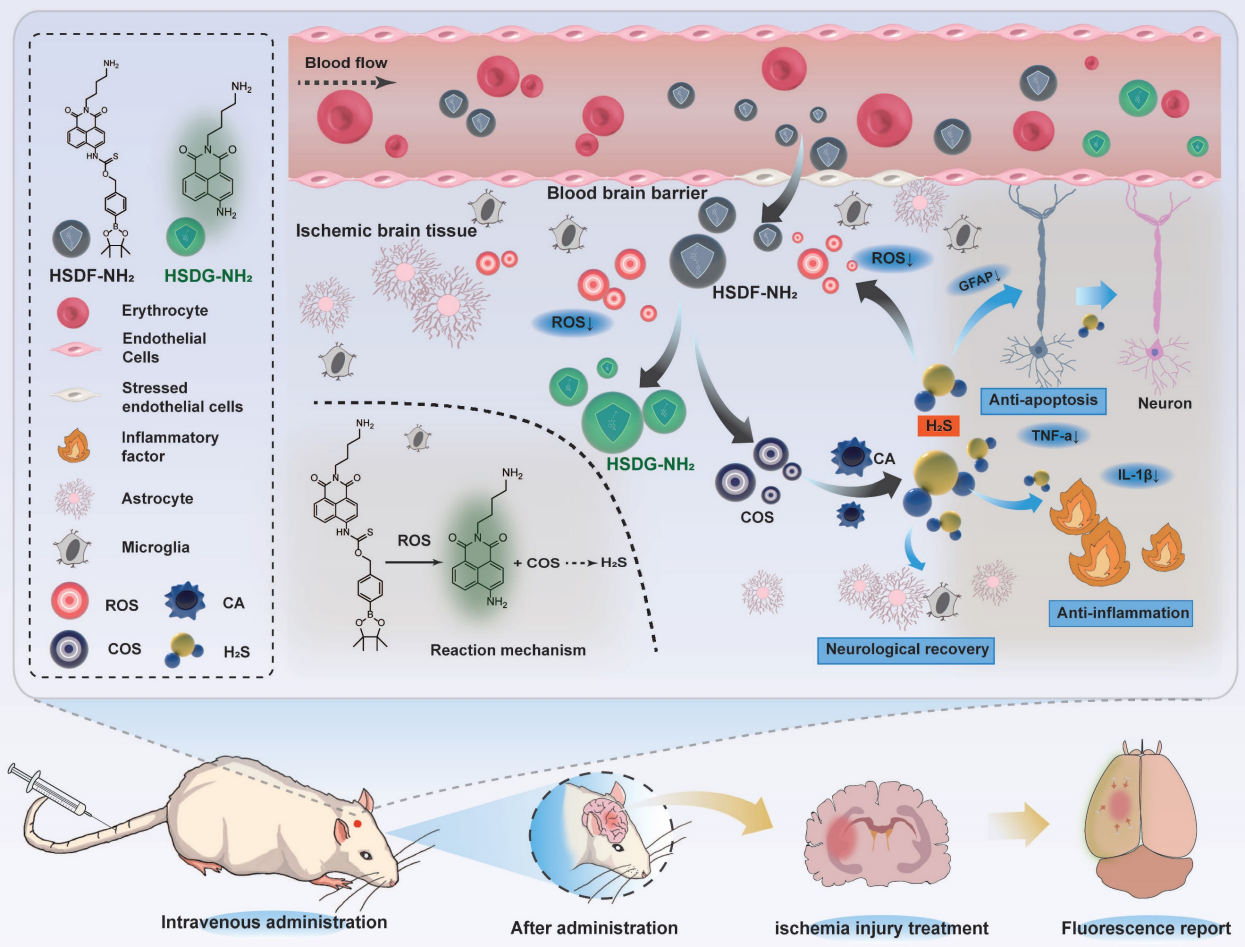
Illustration of the reaction mechanism of the hydrogen sulfide donor and the production of hydrogen sulfide and related pharmacological effect.

Considering these factors, we previously designed a novel fluorescent H_2_S donor (HSD-B), which enabled the visualization and quantification of H_2_S release *in vitro* 48. On this basis, in order to improve the oil-water partition coefficient of HSD-B, a novel H_2_S donor (named as HSDF-NH_2_) was designed using hydrophilic amino groups instead of triphenylphosphine groups (Scheme 1).

The donor combines the ROS-responsive motif with self-reporting fluorescence transition mechanism that operates without consuming H_2_S. This design aims to simultaneously consume ROS, release H_2_S, and visualize and quantify the released H_2_S. We anticipated that HSDF-NH_2_ would be able to penetrate the damaged BBB. In this process, ROS would react with phenylboronic acid pinacol ester to release COS, which would then be hydrolyzed to form H_2_S catalyzed by the enzyme carbonic anhydrase (CA), commonly found in mammals. Concurrently, thiocarbamate-substituted naphthalimides would convert to amine-substituted naphthalimides (designated HSDG-NH_2_), resulting in the restoration of the previously quenched green fluorescence. Finally, PC-12 was used as the cell model to evaluate the neuroprotective effect of HSDF-NH_2_ *in vitro*. We used the rat transient middle cerebral artery occlusion/reperfusion model and evaluated the *in vivo* efficacy and explored the therapeutic mechanism by injecting HSDF-NH_2_ into the tail vein.

## Results and Discussion

### Donor design and characterizations

**Figure 1:**
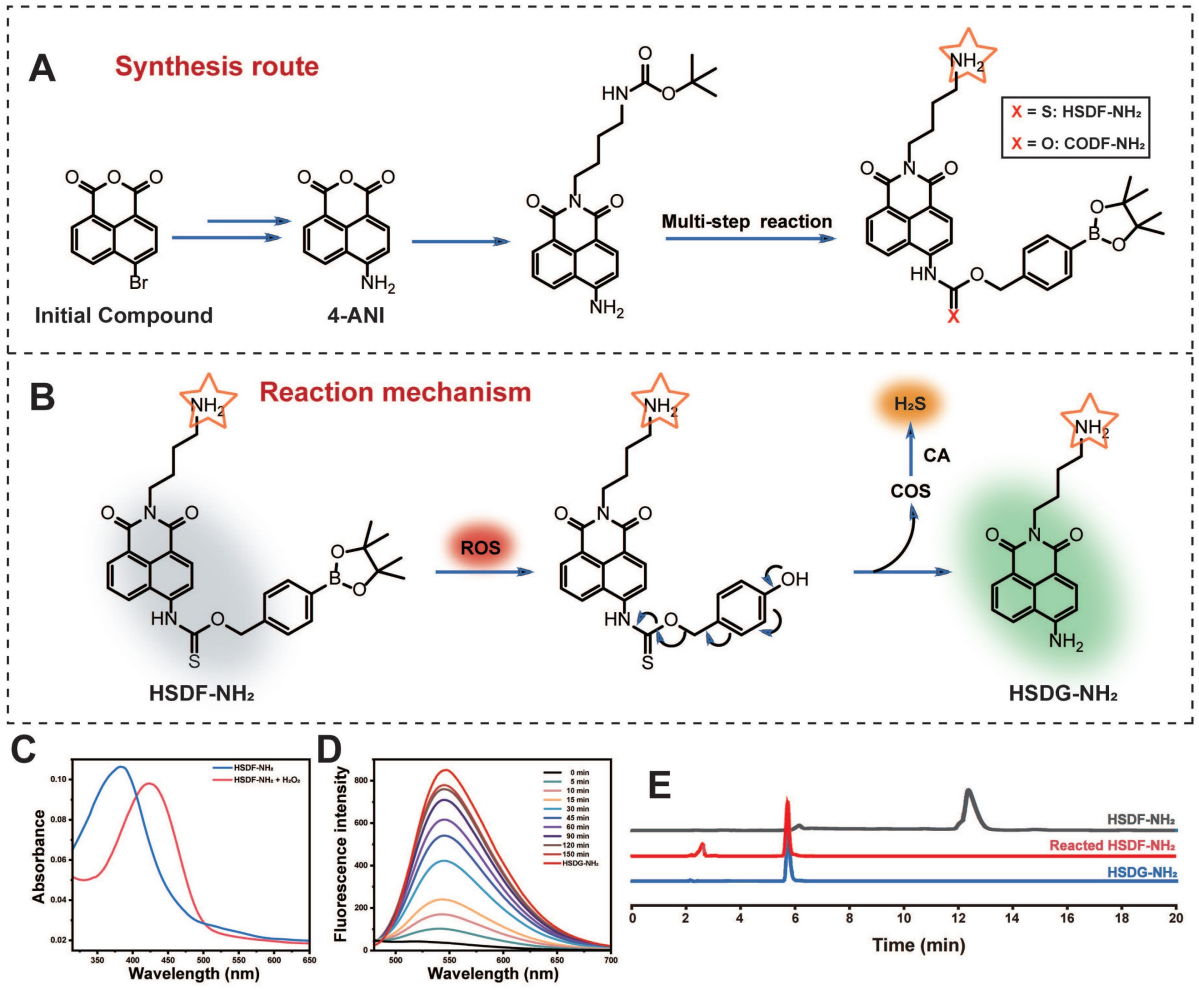
(A) Synthesis of HSDF-NH_2_ and the structure of CODF-NH_2_ (B) The mechanism of H_2_S releasing and monitoring by HSDF-NH_2_ in the presence of ROS. Effects of (C) Absorption spectrum of 10 μM HSDF-NH_2_ reacting with 100 μM H_2_O_2_ at 37 ℃ for 0 min and 150 min. (D) Fluorescence response of HSDF-NH_2_ (10 μM) to H_2_O_2_ (100 μM) and CA (10 μg/mL). HSDG-NH_2_ (10 μM) were used as controls. (E) HPLC traces of the samples: HSDF-NH_2_, HSDF-NH_2_ after reacting with H_2_O_2_ in PBS buffer for 120 min, and HSDG-NH_2_.

Among various fluorescent dyes, naphthalimide is notable for its ease of modification and exceptional optical properties 49. The compound HSDF-NH_2_ was synthesized by linking the phenylboronic acid pinacol ester to the 4-amino-naphthalimide tethered thiocarbamate (Figure 1A). The fluorescence of 4-amino-naphthalimide is quenched by the electron-withdrawing group of thiocarbamate. The ROS-responsive phenylboronic acid pinacol ester acts as a switch for the donor. In regions of stroke infarction, HSDF-NH_2_ is activated by ROS to yield COS, a precursor of H_2_S. Concurrently, the unstable intermediate is converted to HSDG-NH_2_, which exhibits strong fluorescence and facilitates real-time monitoring of H_2_S release. The introduction of an amino group at the N-position of 1,8-naphthalimide enhances the logD value of the donor and satisfies the covalent binding requirements of various designs (Figure 1B).

**Figure 2:**
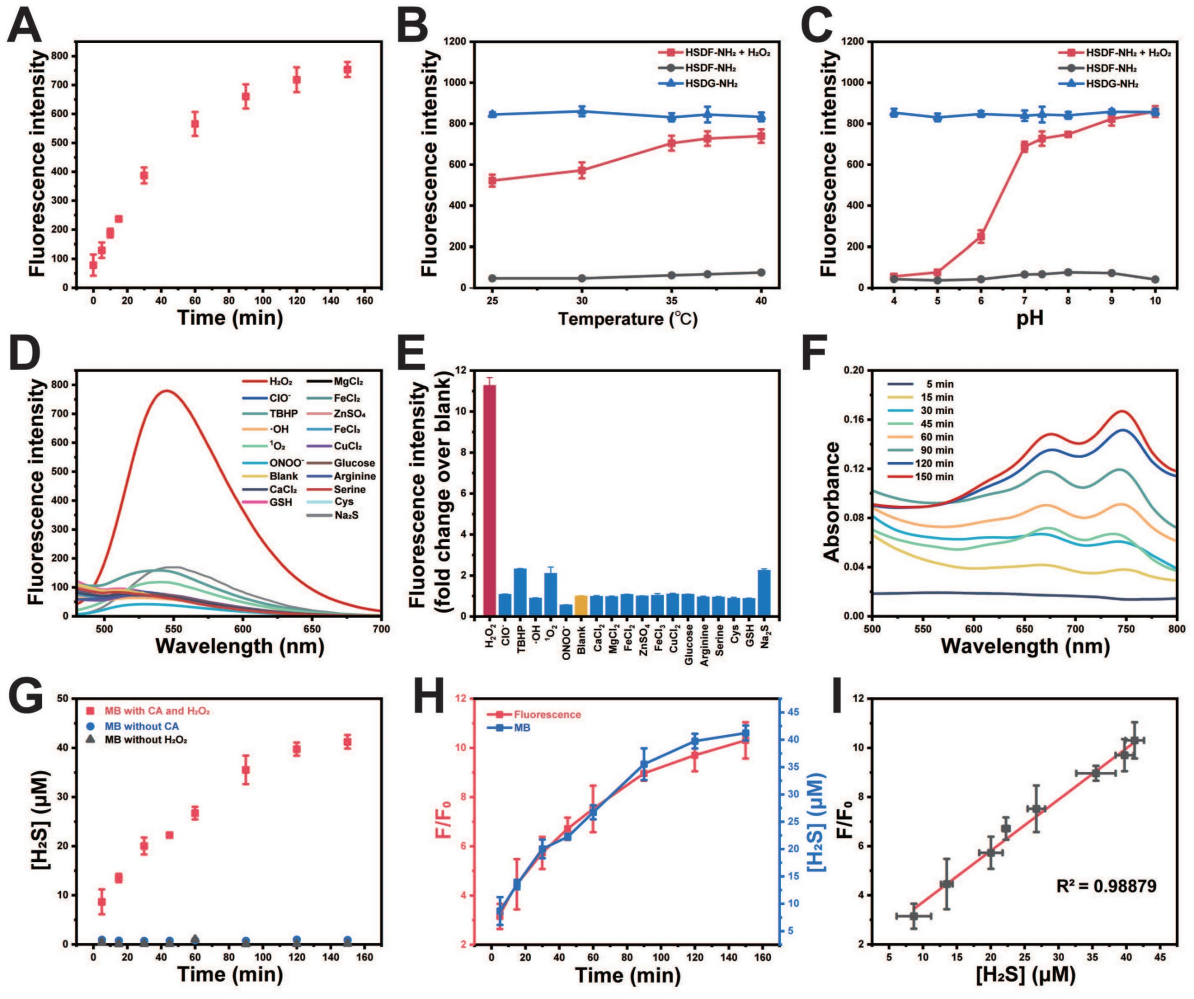
*In vitro* characterizations of HSDF-NH_2_. (A) Time-dependent fluorescence intensities of HSDF-NH_2_ at 565 nm in the presence of H_2_O_2_ (100 μM). (B) Temperature at pH 7.4 and (C) pH at 37 ℃ on the fluorescence of HSDG-NH_2_ and HSDF-NH_2_ reacting with 0 and H_2_O_2_ for 2 h. λex/em = 427/565 nm. (D) Fluorescence intensity at of 10 μM HSDF-NH_2_ toward various species. Deionized water was used as a control. (E) Fluorescence responses of 10 μM HSDF-NH_2_ to various species. (F) UV-Vis absorption spectrum of HSDF-NH_2_ at different time points via MB assay. (G) H_2_S release from HSDF-NH_2_ upon introducing CA and H_2_O_2_ (■), H_2_O_2_ without CA (●) and CA without H_2_O_2_ (▲). (H) Time-dependent fluorescence turn on (red) and H_2_S release (blue) of HSDF-NH_2_. (I) Correlation between fluorescence measurement and MB detection.

The spectral properties and response of the donor HSDF-NH_2_ (10 μM) were studied in phosphate buffer (10 mM, pH = 7.4) as solvent. The maximum absorption of HSDF-NH_2_ is at 385 nm. Upon incubation with H_2_O_2_ (100 μM) at 37 ℃, the maximum absorption peak of the test solvent appeared gradually at 427 nm (Figure 1C). H_2_O_2_ triggers chemoselective cleavage of the boronate-based thiocarbamate protecting group of HSDF-NH_2_ to deliver HSDG-NH_2_ as characterized by its emission spectra (λ_em_ = 565 nm). As shown in Figure 1D, Figure 2A and Figure S3, in the presence of H_2_O_2_ and CA, the emission peak of HSDF-NH_2_ at 565 nm increased concomitantly and almost reached the intensity of HSDG-NH_2_ at 150 min.

The effects of pH and temperature on the reaction between HSDF-NH_2_ and H_2_O_2_ were also investigated. As shown in Figures 2B-C, the reaction remains inert in acidic conditions, whereas the fluorescence intensity significantly increases with rising pH values. In the presence of H_2_O_2_, the aryl-bronic ester C-B bond is oxidized to phenol (C-O bond). Higher pH values are also likely to enhance the ensuing 1,6-elimination that releases COS and HSDG-NH_2_ due to the reactive phenoxide (RO-) form being the dominant species in solution under more basic conditions. Although higher temperatures were favorable for the reaction, HSDF-NH_2_ also demonstrated effective performance under physiological conditions (37 ℃, pH 7.4). The fluorescence intensity of HSDF-NH_2_ after reacting with varying concentrations of H_2_O_2_ for 40 min was recorded (Figure S1). A strong linear correlation between the fluorescent signal and H_2_O_2_ concentration was observed over the range of 0 to 150 μM (Figure S1 inset). To evaluate the selectivity of HSDF-NH_2_ for H_2_O_2_, various analytes, including ROS, inorganic salts, sulfur, and amino acids 50, were tested. As shown in Figures 2D-E, only the addition of H_2_O_2_ resulted in a significant increase in fluorescence, whereas other analytes had negligible effects. These findings indicate that HSDF-NH_2_ exhibits excellent sensitivity and selectivity for H_2_O_2_.

### Methylene blue colorimetric method for H_2_S detection

To ascertain the ability of the donor to release H_2_S in the presence of CA and H_2_O_2_, the widely recognized methylene blue method was employed to quantify H_2_S generation. As shown in Figure 2F, the combination of methylene blue solution with HSDF-NH_2_ solution, H_2_O_2_, and CA exhibited distinct absorption peaks at 670 nm and 745 nm, unequivocally indicating the release of H_2_S by HSDF-NH_2_. Using the established calibration curve (Figure S2), the concentration of H_2_S liberated by HSDF-NH_2_ was quantitatively determined and presented in Figure 2G, revealing a time-dependent release pattern with peak release occurring approximately 150 min after initiation, achieving an efficiency of approximately 30%. The underlying factors contributing to this temporal profile are likely multifaceted, involving the oxidative properties of H_2_O_2_ and the volatility of the gas. Notably, omission of CA from the reaction system notably suppressed the absorbance at 670 nm, underscoring the dependency of HSDF-NH_2_'s H_2_S release on CA during the conversion from COS to H_2_S. Crucially, the robust linear correlation observed between fluorescence measurements and H_2_S quantification via the methylene blue method (R^2^ = 0.988) underscores the reliability of fluorescent readings as optical tools for monitoring COS/H_2_S release dynamics from HSDF-NH_2_ with high temporal resolution (Figure 2H-I).

### The proposed mechanism of H_2_S release from HSDF-NH_2_ with self-reporting fluorescence

The proposed mechanism detailing H_2_S release from HSDF-NH_2_, along with its self-reporting fluorescence in the presence of ROS, is illustrated in Figure 1B. Initially, HSDF-NH_2_ undergoes partial hydrolysis to boronic acid upon exposure to water, followed by oxidation triggered by hydrogen peroxide. This sequential process yields the fluorescent amino compound HSDG-NH_2_ and releases COS. The caged COS subsequently undergoes conversion to H_2_S in the presence of CA. The confirmation of these compounds was achieved using HPLC and high-resolution mass spectrometry (HRMS), as depicted in Figure 1E and Figure S4.

### Neuroprotective effect of HSDF-NH_2_ in tMCAO/R rats

**Figure 3:**
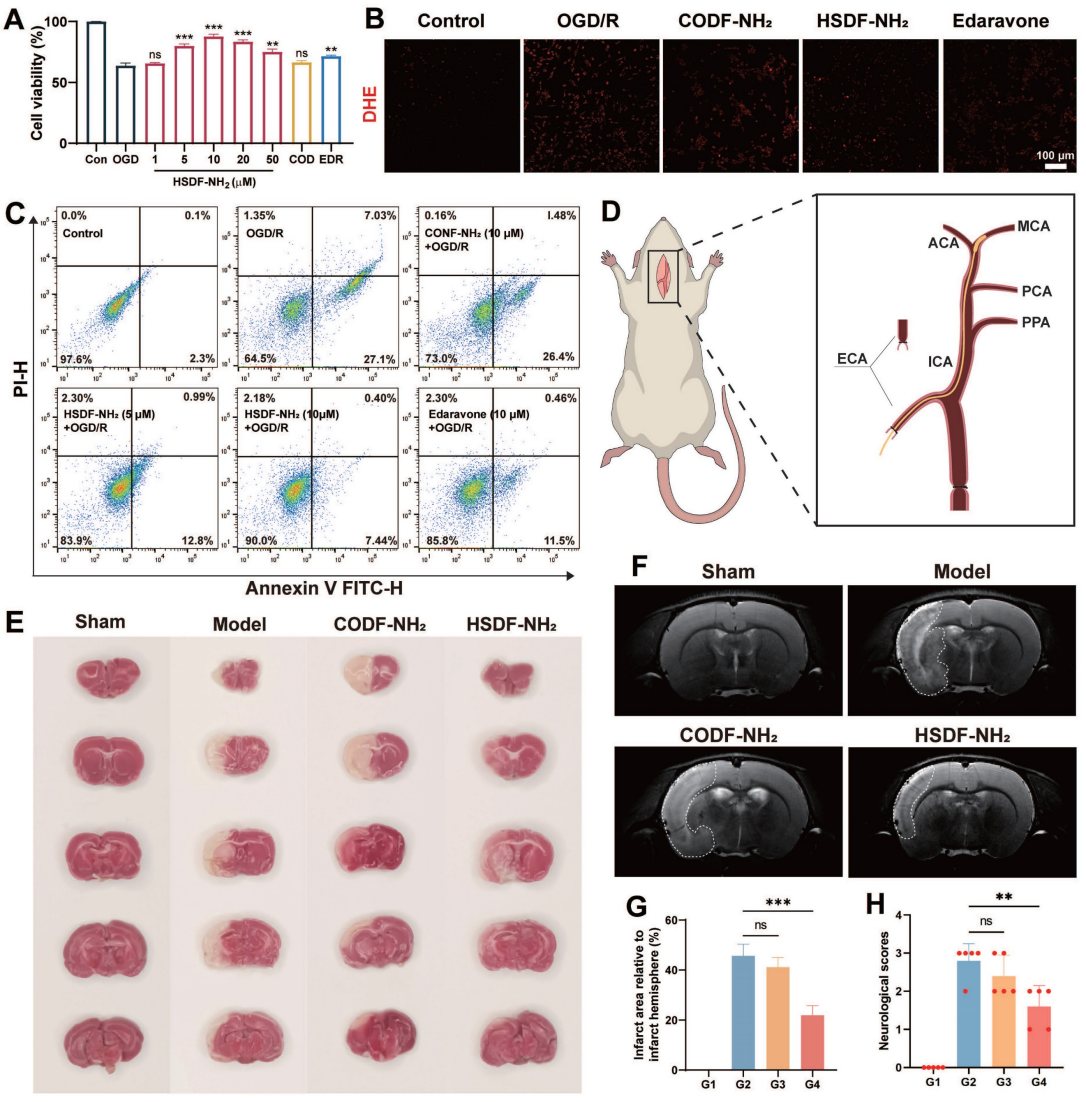
(A) Cell viabilities of OGD-treated PC-12 cells incubated with HSDF-NH_2_ for 24 h. Pretreated PC-12 cells with different concentrations of HSDF-NH_2_ (0, 1, 5, 10, 20, and 50 μM), CODF-NH_2_ (COD, 10 μM), or EDR (edaravone, 10 μM, positive control). Data are presented as means ± SD, n = 3. **P < 0.01; ***P < 0.001. (B) Representative images of the DHE fluorescence in PC-12 cells treated with OGD/R or different groups (Scale bar: 100 μm). (C) Flow cytometry analysis of hypoxia-induced apoptosis of PC-12 cells pretreated with PBS, various doses of HSDF-NH_2_, CODF-NH_2_ or Pro. (D) Schematic diagram of the model of tMCAO/R established by the currently accepted suture embolic method (ECA: External carotid artery; ICA: Internal carotid artery; PPA: Paramedian pontine arteries; PCA: Posterior cerebral artery; MCA: Middle cerebral artery; ACA: Anterior cerebral artery). (E) The infarct area of tMCAO/R rats with different treatment, brain slices were stained with TTC at 72 h post reperfusion. (F) The infarct area of tMCAO/R rats treated with different drugs, monitored by MRI at 4th day post reperfusion. (G) The quantified results of TTC staining in E (G1: Sham; G2: Model; G3: CODF-NH_2_; G4: HSDF-NH_2_). Data are presented as means ± SD, n = 3, ***P < 0.001. (H) The neurological assessment of tMCAO/R rats treated with different drugs. Data are presented as means ± SD, n = 5, **P < 0.01.

The protective effect of HSDF-NH_2_ on I/R injury in the hypoxia/reoxygenation (H/R) model of PC-12 cells was investigated. The cell viability assay demonstrated that HSDF-NH_2_ exhibited a dose-dependent protective effect on OGD-insulted PC-12 cells (Figure 3A). Excessive ROS accumulation in the infarct microenvironment exacerbates injury 51; therefore, intracellular ROS levels can serve as an indirect indicator of oxidative stress following cellular damage. In this study, we utilized the ROS probe dihydroethidium (DHE) to monitor and analyze intracellular ROS levels in PC-12 cells after various treatments (Figure 3B and S7). Compared to untreated control cells, OGD/R-treated cells exhibited significantly enhanced DHE red fluorescence signals, indicating an abnormal increase in ROS levels. Treatment with HSDF-NH_2_ and edaravone significantly inhibited the ROS elevation, confirming the synthesized donors' effective ROS scavenging capability at the cellular level. Flow cytometry analysis further elucidated the protective mechanisms of HSDF-NH_2_ against I/R injury (Figure 3C). HSDF-NH_2_ significantly reduced the percentage of apoptotic and necrotic PC-12 cells induced by H/R injury, demonstrating an anti-apoptotic effect comparable to that of Pro. These results provide compelling evidence that HSDF-NH_2_ is a potent H_2_S donor, offering cellular protection from oxidative stress.

The neuroprotective effect of HSDF-NH_2_ was assessed using the rat transient middle cerebral artery occlusion/reperfusion (tMCAO/R) model, established via the widely accepted suture embolic method (Figure 3D) 52. Primary neurological scores 53 were evaluated 24 h post-treatment, revealing that the HSDF-NH_2_ group scored 1.6 points, in contrast to the CODF-NH_2_ and model groups, which scored 2.4 and 2.8 points, respectively. This indicates that the HSDF-NH_2_ group experienced the most significant recovery in neurological function (Figure 3H). TTC staining was employed to distinguish between infarcted and normal brain tissue, with infarcted regions appearing white and normal regions red. On day 3 post-treatment, TTC staining demonstrated substantial brain recovery in the HSDF-NH_2_ group (Figure 3E). Specifically, the infarcted area was reduced to 21.93% in the HSDF-NH_2_ group, compared to 45.7% in the model group (Figure 3G). These findings were corroborated by MRI imaging, which also indicated a reduction in infarct size (Figure 3F).

### Fluorescence imaging of H_2_S release from HSDF-NH_2_ *in vitro* and *in vivo*

**Figure 4:**
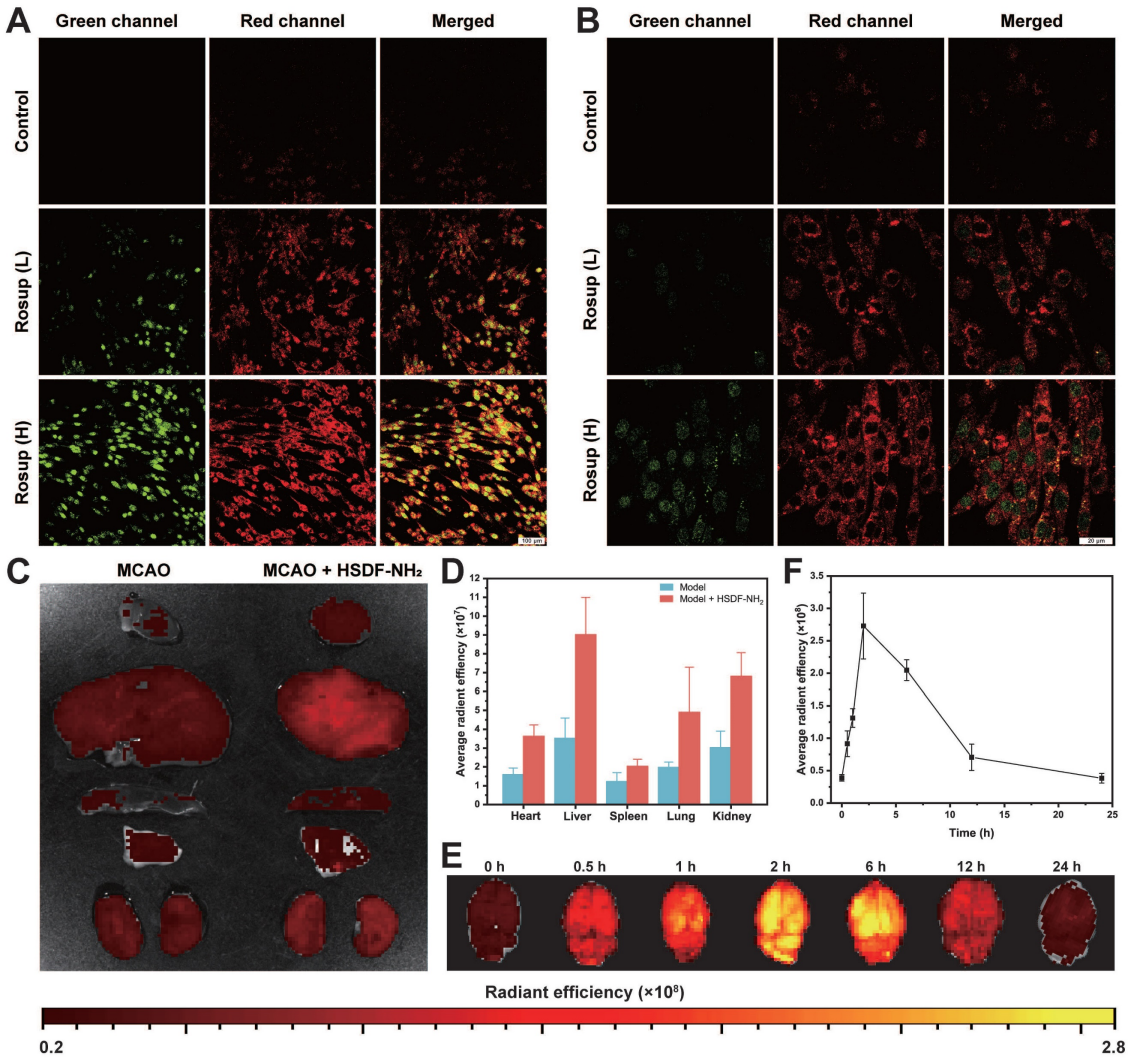
(A) 20× and (B) 63× Confocal microscopy images of PC-12 cells after different treatments. Cells were first incubated with HSDF-NH_2_ (10 μM) and Cy-NO_2_ (10 μM) for 60 min. After removal of excess HSDF-NH2 and Cy-NO2, PBS (control), low-dose Rosup at 50 μg/mL (Rosup (L)), or high-dose Rosup at 100 μg/mL (Rosup (H)) was added. Fluorescence images were acquired after 30 min (20× mirror scale bar: 100 µm; 63× mirror scale bar: 20 µm). Representative IVIS images (C) and quantification (D) of main organs after intravenous administration of different drugs for 2 h. *Ex vivo* fluorescence images (E) and quantification (F) of H_2_S release in brains collected from t/MCAO rats at different time points after treatment with the same dose of HSDF-NH_2_. Data are presented as means ± SD, n = 3.

The fluorescence properties of HSDF-NH_2_ were further evaluated for potential biomedical imaging applications. In cell culture experiments, Rosup was used to induce the generation of H_2_O_2_ and subsequent release of H_2_S. To monitor H_2_S production within the cells, Cy-NO_2_ was employed as a fluorescent probe 54. In the absence of Rosup, no red fluorescence signal from Cy-NO_2_ was detected, indicating that H_2_S was not produced (Figures 4A-B). However, after 30 min of incubation with Rosup, a dose-dependent increase in fluorescence signals was observed in both the donor and probe channels. Analyzed at the cellular level, HSDF-NH_2_ exerted the best effect at the level of ROS scavenging, protection of neuronal cells, and reduction of apoptosis. And as a control, CODF-NH_2_ group could observe a certain but weak neuroprotective effect, which confirmed that the direct quenching of ROS by the borate structure could not maximize the realization of the protective effect, and it was obvious that the release of H_2_S was the key. And we also found that Rusup-treated cells do not emit fluorescence in the Cy-NO_2_ channel in the absence of HSDF-NH_2_ presence, which demonstrates that Cy-NO_2_ is stable under the experimental conditions without the introduction of a donor and can accurately represent H_2_S release (Figure S6). These results confirm that HSDF-NH_2_ successfully delivered H_2_S and provided enhanced green fluorescence, enabling real-time monitoring of H_2_S release in living cells.

Given that the oil-water Distribution coefficient (logD) is a critical parameter for evaluating the capacity of a small molecule to cross the blood-brain barrier 55, we have preliminarily estimated the logD value of HSDF-NH_2_ to be approximately 2.35. Notably, the differential fluorescence intensities between HSDF-NH_2_ and its cleaved product (HSDG-NH_2_) upon H_2_S release facilitated the monitoring and imaging of H_2_S release in t/MCAO rats. A single dose of HSDF-NH_2_ at 4 mg/kg was administered via tail vein injection in tMCAO/R rats, and subsequent fluorescence changes were observed through *ex vivo* imaging. The results indicated that the green fluorescence, which signifies the presence of HSDG-NH_2_ in the brain after the conversion of HSDF-NH_2_, increased gradually, peaking at 2 h before declining and becoming weak at 24 h (Figure 4E-F). This observation suggests that HSDG-NH_2_ is metabolized and cleared from the brain over time. Furthermore, *ex vivo* imaging of various organs following HSDF-NH_2_ administration in tMCAO/R rats revealed that HSDF-NH_2_ is predominantly metabolized by the liver and kidneys (Figure 4C-D). However, HSDF-NH_2_ presents certain limitations for *in vivo* imaging and quantification, primarily due to autofluorescence in live animals and the insufficient emission wavelength of HSDG-NH_2_.

### Behavior tests and mechanisms underpinning therapeutic effects of HSDF-NH_2_ on tMCAO/R

**Figure 5:**
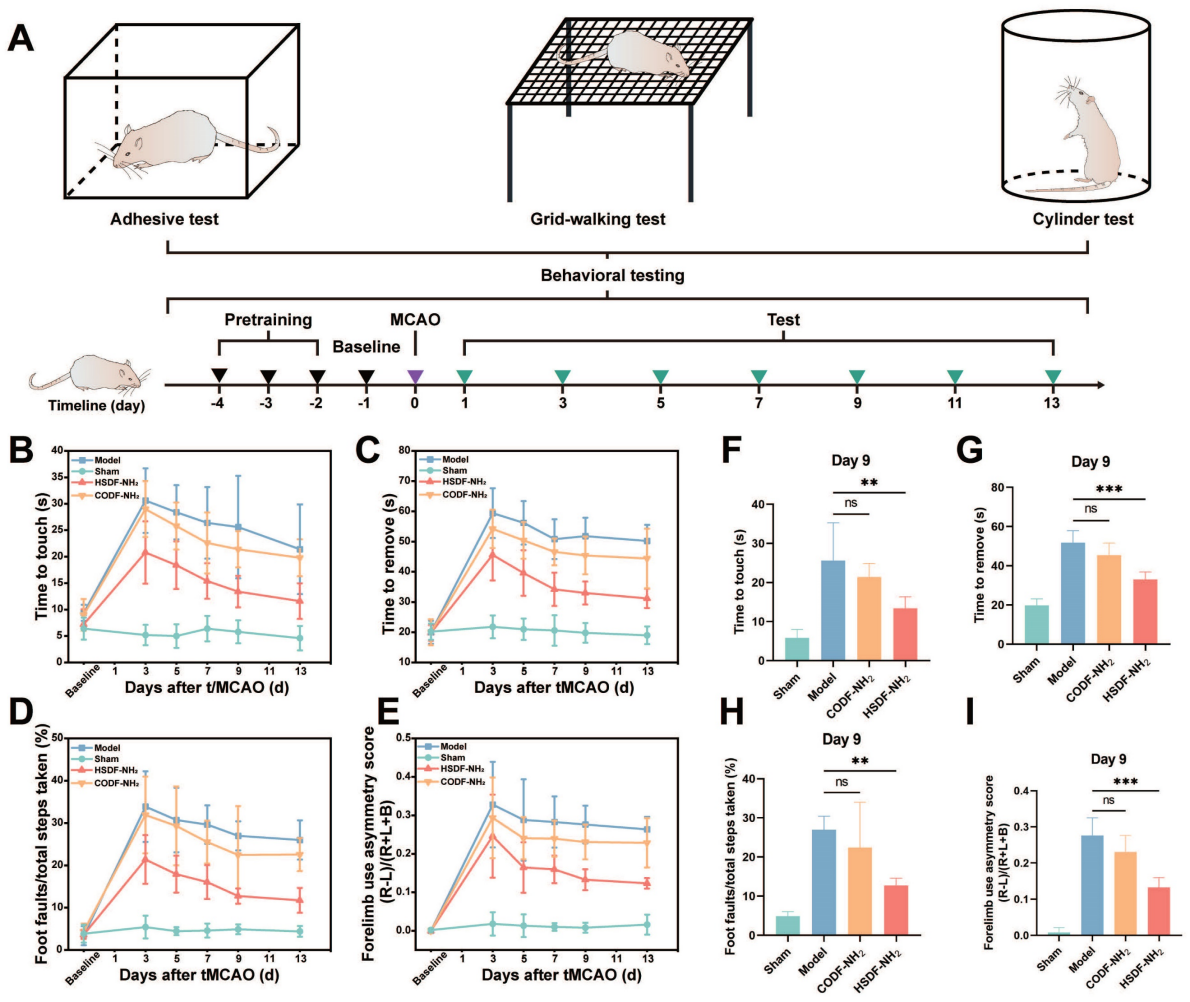
(A) Schematic illustration of the timeline of behavioral test. Functional recovery was evaluated by (B-C) the adhesive test, (D) grid-walking test and (E) the cylinder test at a series of time points after tMCAO/R. Results of the (F-G) adhesion test, (H) grid walking test, and (I) cylinder test on day 9 after tMCAO/R. Data are presented as means ± SD, n = 5.

As shown in Figure 5A, the tMCAO/R model was successfully established following the pretraining of rats. Behavioral assessments were conducted on days 1, 3, 5, 7, 9, 11, and 13. In the adhesive removal test (Figures 5B-C, F-G), rats in the ischemic model group exhibited severe behavioral deficits. However, 9 days post-treatment with HSDF-NH_2_, this behavioral asymmetry was significantly ameliorated, indicating a substantial therapeutic effect of HSDF-NH_2_. In the grid-walking test, the model groups demonstrated increased contralateral foot faults, whereas these faults were significantly reduced in the HSDF-NH_2_-treated groups (Figures 5D, H). The cylinder test further revealed that tMCAO/R induced a higher rate of asymmetry, which was significantly improved with HSDF-NH_2_ treatment (Figures 5E, I), underscoring the motor-functional neurological recovery facilitated by HSDF-NH_2_.

**Figure 6:**
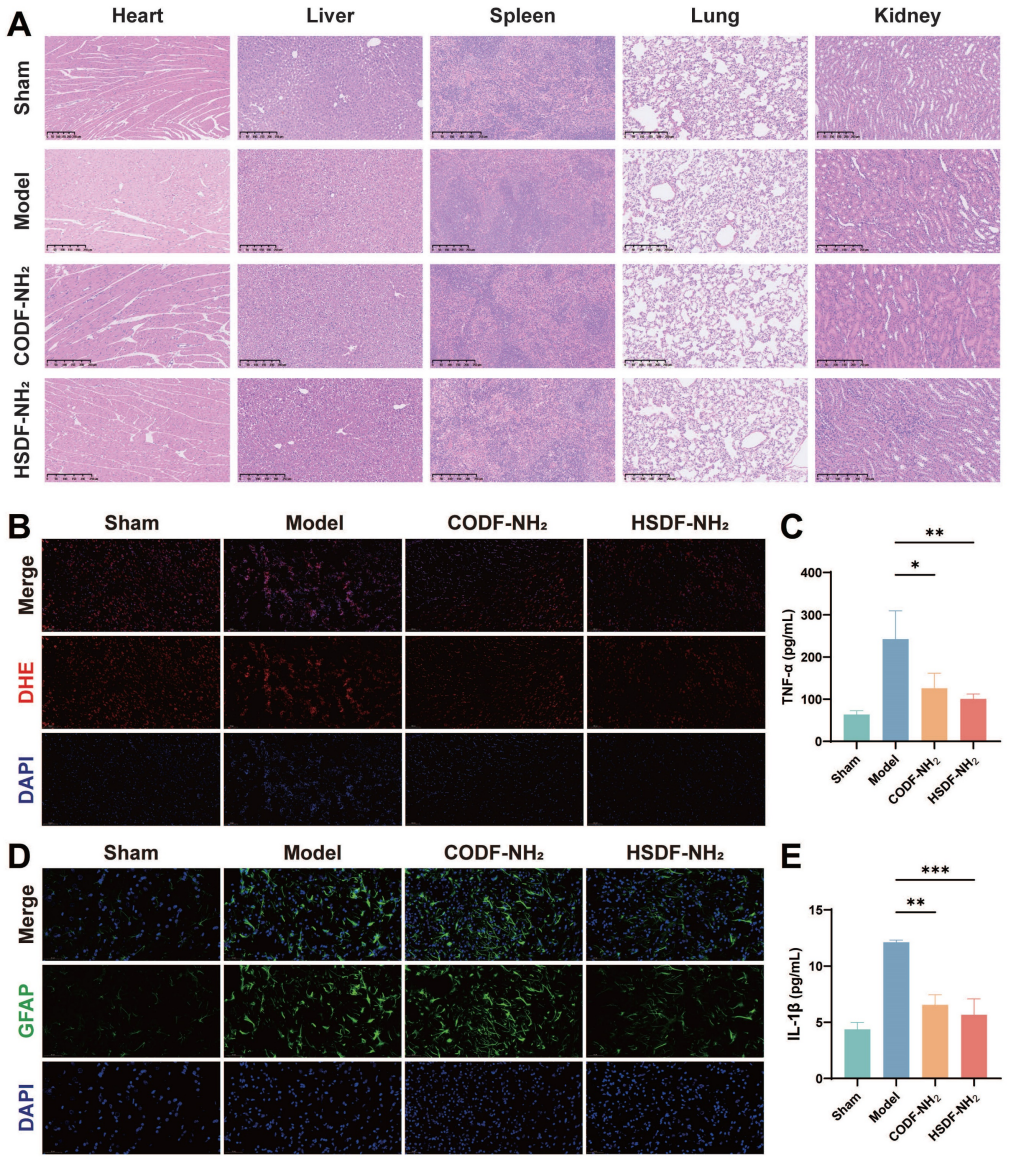
(A) Representative images of main organs with H&E staining after the tMCAO/R rats were treated with different drugs (Scale bar, 250 μm). (b) Representative DHE staining indicating the ROS level in neurons in penumbra of tMCAO/R rats with different treatments. DHE (red), DAPI (blue), (Scale bar: 100 μm). (d) Glial scars (GFAP) level in infarct sites of various groups 14 days after different treatments. GFAP (green), DAPI (blue), (Scale bar: 50 μm). The expression of proinflammatory cytokines including (c) TNF-α and (E) IL-1β decreased. Data are presented as means ± SD, n = 3, *P < 0.05, **P < 0.01, ***P < 0.001.

We then investigated the capability of HSDF-NH_2_ to modulate the proinflammatory microenvironment and rescue damaged neurons. HSDF-NH_2_ significantly reduced the expression of pro-inflammatory cytokines TNF-α and IL-1β (Figures 6C, E). To assess oxidative stress levels in the lesion, we utilized the DHE probe to detect changes in ROS content (Figure 6B). The results demonstrated that HSDF-NH_2_ administration markedly decreased ROS levels in the ischemic semi-dark band, thereby reducing neuronal oxidative stress compared to the model group. Additionally, we examined the therapeutic effects of HSDF-NH_2_ on glial scar formation. 14 days post-treatment, we observed reduced GFAP expression in the infarcted hemisphere in the HSDF-NH_2_ group (Figure 6D). Given the additional ROS generated during reperfusion, studies utilizing reperfusion models of brain injury could yield more clinically relevant results. Whether HSDF-NH_2_ can exert beneficial effects in I/R models warrants further investigation.

### *In vitro* and *in vivo* biocompatibility of HSDF-NH_2_

HSDF-NH_2_ did not exhibit significant cytotoxicity at concentrations up to 20 μM in normal PC-12 cells (Figure S5), indicating high cytocompatibility. Furthermore, blood routine examinations of both sham and model rats post-injection of HSDF-NH_2_ revealed no noticeable abnormalities (Figure S8), demonstrating its low toxicity *in vivo*. Additionally, histological H&E staining analyses of the heart, kidney, liver, lung, and spleen tissues showed no apparent variations (Figures 6A and S9), further underscoring the favorable biological safety profile of HSDF-NH_2_.

## Conclusions

In conclusion, we have designed and synthesized a novel ROS-responsive H_2_S donor, HSDF-NH_2_, which not only scavenges ROS, but also releases H_2_S and activates fluorescence. This compound significantly increased the viability of OGD/R-injured PC-12 cells *in vitro*. Furthermore, its cytoprotective effects were successfully translated to an *in vivo* tMCAO/R rat model. HSDF-NH_2_ effectively penetrates the blood-brain barrier and exhibits therapeutic effects by reducing infarct volume, decreasing apoptosis, and mitigating oxidative stress. As a result, neurological function was notably improved in rats treated with HSDF-NH_2_. Overall, HSDF-NH_2_ introduces new strategies for the treatment of ischemic stroke.

## Experimental section

### Materials and instruments

All chemicals were obtained from commercial suppliers and used without additional purification. Roswell Park Memorial Institute (RPMI-1640) medium, penicillin and streptomycin were purchased from Thermo Fisher Scientific (Massachusetts, U.S.A). Trypsin was purchased from NCM Biotech (Suzhou, China). ROS Assay Kit was purchased from BIOESN (Shanghai, China). Fetal bovine serum (FBS) and Annexin V-FITC apoptosis detection kit was purchased from Pricella (Wuhan, China). The monofilament nylon threads were purchased from Meyue (Changsha, China). 2,3,5-Triphenyltetrazolium chloride staining solution (2%) was purchased from Solarbio (Beijing, China). ^1^H NMR and ^13^C NMR spectra were recorded on a Bruker AV-400 or 600 MHz spectrometer. The NMR data were processed by software Mest Re-Nova (Ver.14.0.0.23239, Mestrelab Research S.L.). Chemicals shifts were referenced to the residue solvent peaks and given in ppm. High-resolution mass spectra (HRMS) were obtained using a Q-STAR Elite ESI-LC-MS/MS spectrometer. UV-Vis absorption spectra were acquired on a JASCO V-730 spectrophotometer. Fluorescence emission spectra were acquired on a PerkinElmer LS55 and Edinburgh FS5 fluorescence spectrophotometer. Samples for absorption and fluorescence emission measurements were contained in 1×1 cm quartz cuvettes (3.5 mL volume).

### Synthesis and characterization of HSDF-NH_2_, CODF-NH_2_ and HSDG-NH_2_

The synthetic route of HSDF-NH_2_, CODF-NH_2_ and HSDG-NH_2_ were exhibited in supplementary information (Supplementary Data S1). The structure of donor was verified by ^1^H NMR, ^13^C NMR and HRMS (Figure S10-S30).

### General methods of UV-Vis absorption and fluorescence spectra

Unless otherwise noted, all the spectral measurements were carried out in 10 mM phosphate buffer (pH 7.4) according to the following procedure in triplicate. Typically, 35 mL of HSDF-NH_2_ (10 μM) solution was prepared and added with CA (10 μg/mL) and H_2_O_2_ (final concentration of 100 μM). The resulting mixture was well shaken and placed at 37 ℃ before measurement.

### Preparation of various solutions for selectivity analysis

A stock solution of 10 mM HSDF-NH_2_ was prepared in DMSO. The fluorescence response of HSDF-NH_2_ (10 μM) was performance in 3 mL PBS (10 mM pH = 7.40). The solutions containing different ROS and thiol nucleophiles (Cys, GSH, Na_2_S) were prepared by dissolving the corresponding compounds in ultrapure water (final concentration of 100 μM). ClO^-^, H_2_O_2_, ONOO^-^, ^1^O_2_ and TBHP were generated according to the previous report 56. •OH was generated from the reaction between Cu^2+^ and ascorbate 57. Metal ions (Ca^2+^, Mg^2+^, Zn^2+^, Fe^2+^, Fe^3+^, Cu^2+^), glucose and thiol nucleophiles were also prepared in appropriate concentrations. The final concentration of metal ions is 30 μM, and the final concentration of glucose or amino acid is 1.5 mM. And CaCl_2_, ZnSO_4_, FeCl_2_, FeCl_3_, CuCl_2_ were used for the metal ions solution preparation. Reaction time was set to be 120 min for all reagents.

### HPLC measurement

HPLC was conducted using the Agilent 1260 Infinity II system. The analysis utilized a SuperLuC18-AQ5u column (4.6 mm × 250 mm, 5 μm) with a mobile phase composed of acetonitrile and 25 mM ammonium acetate buffer (8:2, v/v) at a flow rate of 1 mL/min and detection at 254 nm. The reaction mixture of HSF-NH_2_ and H_2_O_2_ in PBS (10 mM, pH 7.4) served as the sample for measurement.

### Methylene blue H_2_S release assay

Each assay described was performed in triplicate in a one-dram vial containing 1.165 mL PBS buffer (pH = 7.4), 300 μL Zn(OAc)_2_ solution (1% w/v), 20 μL HSDF-NH_2_ solution (10 mM in DMSO), H_2_O_2_ solution, and 300 μL CA solution (0.5 mg/mL). The final concentrations were 133 μM HSDF-NH_2_, 1 mM H_2_O_2_, and 100 μg/mL CA. At predetermined timepoints, 1.8 mL was removed from each reaction vial and diluted with 600 μL FeCl_3_ solution (30 mM in 1.2 M HCl), followed by 600 μL N, N-dimethyl-p-phenylenediamine solution (20 mM in 7.2 M HCl). The MB reaction was conducted for 90 min, and the absorbance at 670 nm of the resulting solution was measured with an UV-Vis spectrometer. The concentration of H_2_S in each sample was determined from a calibration curve of Na_2_S, which was generated by plotting the H_2_S concentration against the measured absorbance.

### LogD measurement procedure

Initially, n-octanol served as a lipid solvent to dissolve HSDF-NH_2_ of different concentrations, and the corresponding standard calibration curves were constructed using UV-visible spectroscopy. Subsequently, the PBS buffer solution (pH 7.4) was combined with n-octanol in a 1:1 volumetric ratio, followed by the introduction of a predetermined amount of the probe while agitating. After thorough mixing for 120 minutes, the mixture was left undisturbed to allow for phase separation of the n-octanol, and the UV absorbance was then measured. Subsequently, the concentration of the probe in the oily phase (C_oil_) was determined using the established standard curve. The probe's concentration in the aqueous phase (C_water_) was quantitatively deduced based on the principle of mass conservation. The partition coefficient (LogD) was calculated as the logarithm of the ratio of C_oil_ to C_water_. The final LogD values were derived from the mean of triplicate measurements.

### Cell culture and oxygen-glucose deprivation/reoxygenation (OGD/R) Model

PC-12 cell line was purchased from the iCell Bioscience Inc (Shanghai, Chian). The OGD/R model for PC-12 cells was developed using the following steps: The ischemia was simulated by replacing the high glucose RPMI-1640 medium with 12% FBS with a glucose-free RPMI-1640 medium. After incubating for 12 h at 37 ℃ in an anoxic environment, the cells were transferred back to a conventional incubator for 24 h with a fresh high glucose medium to mimic reperfusion. PC-12 cells maintained under normoxic conditions with high glucose RPMI-1640 medium served as control. Five groups of PC-12 cells were prepared: the normal (Con) group, the OGD/R model group, the HSDF-NH_2_ pretreatment groups (HSDF-NH_2_, 1-50 μM), the CODF-NH_2_ group (10 μM, COD), and the propranolol pretreatment group (10 μM, EDR) as the positive control.

### Cytotoxicity assay

The MTT assay was employed to assess the cytotoxicity of the compound HSDF-NH₂. Briefly, PC-12 cells were seeded in 96-well plates at a density of approximately 1 × 10⁴ cells per well and incubated for 24 h. Subsequently, fresh medium containing the test or control compounds at varying concentrations was added to the PC-12 cells. Following a 24 h incubation with the compounds, the cells were washed twice with PBS, and 100 μL of MTT solution (0.5 mg/mL) was added to each well. After an additional 4 h incubation at 37 ℃, the medium was removed and 100 μL of DMSO was added to dissolve the formazan crystals. The absorbance of each well was then measured at 570 nm using a plate reader.

### ROS scavenging capability of HSDF-NH_2_ in OGD/R model

To measure the ROS levels in cells subjected to OGD/R, DHE, which converts into a highly red fluorescent compound upon oxidation by ROS, was used to detect intracellular ROS formation. Briefly, PC-12 cells were plated in 24-well plates and subjected to OGD for 12 h. The cells were then cultured in complete medium containing various compounds for 24 h. After washing the cells with DPBS, they were incubated with medium containing DHE (10 μM) for 30 min. Fluorescence images were captured using a confocal laser scanning microscope (LSM800, Zeiss, Germany).

### *In vitro* H_2_S release and fluorescence imaging in PC-12 cells

PC-12 cells were seeded and cultured according to the procedures outlined in section 2.7. The cells were initially treated with HSDF-NH₂ (10 μM) and Cy-NO₂ (10 μM) for 60 min. Following the removal of excess HSDF-NH₂ and Cy-NO₂, the cells were incubated in fresh medium containing either PBS (control), low-dose Rosup (A compound mixture of oxidative reagents with a concentration of 50 mg/ml, usually used as a positive control reagent for reactive oxygen species.) at 50 μg/mL (Rosup (L)), or high-dose Rosup at 100 μg/mL (Rosup (H)) for an additional 60 min. After fixation, the cells were observed and imaged using a confocal laser scanning microscope. The red channel of Cy-NO₂ was recorded at 700-730 nm with excitation at 673 nm, while the green channel of HSDF-NH₂ was recorded at 520-570 nm with excitation at 430 nm.

### Cell apoptosis assay

The effect of HSDF-NH₂ on apoptosis in PC-12 cells was evaluated using an Annexin V-FITC apoptosis detection kit. Briefly, PC-12 cells were seeded in 24-well plates at a density of 1 × 10⁵ cells per well. Following treatment with or without HSDF-NH₂ or edaravone for 24 h, the cells were stained with Annexin V-fluorescein isothiocyanate (FITC) in binding buffer for 15 min at room temperature. The cells were then labeled with propidium iodide (PI), and apoptotic cells were assessed using a flow cytometer (BD FACSCanto).

### Animals

The procedures of animal experiments in this study were approved by the Jinan University (Guangzhou, China). Adult male Sprague-Dawley rats (10-12 weeks old, 200-250 g) were purchased from the Guangdong medical laboratory animal center and housed in standard cages under standard conditions. All animals were acclimatized for one week before use.

### Preparation of the rat tMCAO/R model

The rat transient middle cerebral artery occlusion/reperfusion (tMCAO/R) model was established using a previously reported monofilament method 53, 58. Briefly, a silicone-coated nylon thread (0.32 ± 0.02 mm, catalog number M8507, Changsha Meyue) was inserted into the middle cerebral artery via the ipsilateral external carotid artery to induce occlusion. After 2 h, the thread was removed, and the external carotid artery was ligated to achieve left cerebral ischemia-reperfusion injury in rats. Throughout the procedure, health and humane care were rigorously maintained.

### Evaluation of neurological scores

After 1 h of reperfusion, model rats were randomly assigned to three groups. These groups received intravenous injections of saline (Model), 4 mg/kg of HSDF-NH_2_, and 4 mg/kg of CODF-NH_2_, respectively. Neurological assessments 53 were conducted 24 h post-administration, employing a scoring system as follows: 0, indicating normal and active condition; 1, denoting inability to fully extend the right forepaw; 2, indicating circling towards the right side; 3, indicating inability to stand up and falling towards the right side; and 4, indicating absence of spontaneous movement.

### Neuroprotection effect evaluated with TTC

Brains of MCAO/R rats were collected 72 h post-administration and sliced, immersed in 0.25% 2,3,5-Triphenyltetrazolium chloride (TTC) dye at 37 ℃ for 30 min 6. The infarct area was quantified by ImageJ.

### Behavior tests

The cylinder test, grid-walking test and adhesive removal test were carried out to evaluate the repair of sensorimotor functions. Before surgery, we pre-trained the rats for 3 days continuously. Behavioral tests were then performed at 3th, 5th, 7th, 9th and 13th day post stroke. For the cylinder test, rats were individually placed inside a transparent cylinder measuring 35 cm in height and 15 cm in diameter. The behavior of each rat was observed for 5 min, and the number of contacts made with the cylinder walls using the left forepaw (L), right forepaw (R), or both forepaws (B) was recorded. The asymmetric rate was calculated as (L - R)/(L + R + B) × 100 (%) 59. For grid-walking task, slightly modified from previously reported literature 60, an elevated grid containing square (5× 5 cm^2^) wire mesh was employed. Every rat was placed onto the wire grid to move freely until reaching at least 100 steps with the left forelimb. The numbers of stepping errors and non-faults for both limbs were recorded. The result was then analyzed with the formula reported previously 61. For the adhesive test, a 10 × 10 mm^2^ sticker was placed onto the paralyzed forepaw of the rat. The rat was then returned to its cage. The time taken for the rat to first contact the sticker was recorded as the “time to touch”, and the duration required for the rat to successfully remove the sticker was recorded as the “time to remove” 6.

### MRI imaging *in vivo*

The tMCAO/R rats with different treatment were anaesthetized with isoflurane. To monitor the infarct area, T2-weighted coronal images of the brain were recorded with 9.4 T MR scanner for small animal imaging system (BioSpec 94/30 USR, Burke, Germany) on day 4 post reperfusion. The acquisition parameters for T2-weighted MRI imaging: TR = 2500.0 ms, TE = 33.0 ms, Slice thickness = 0.8 mm. Images were analyzed using RadiAnt DICOM Viever software (Medixant, Poznan, Poland).

### Fluorescence imaging of H_2_S release in tMCAO/R rat models

To study the metabolism and biodistribution of HSDF-NH_2_ in rats with tMCAO/R, rats received a single intravenous injection of HSDF-NH_2_ (4 mg/kg) at 1 h post ligation. Rats in control group were injected with saline. At 2 h post injection, rats were euthanized and major organs including heart, liver, spleen, lung, and kidneys were collected for *ex vivo* imaging (IVIS Spectrum PerkinElmer, U.S.A).

To evaluate the release of H_2_S in living rats with tMCAO/R by *in vivo* imaging, rats received a single intravenous injection of HSDF-NH_2_ (4 mg/kg) at 1 h post ligation. At each defined time point (0, 0.5, 1, 2, 6, 12 and 24 h post injection), rats were euthanized and brains were isolated for *ex vivo* imaging using the IVIS Spectrum (PerkinElmer, U.S.A) in a fluorescence mode.

### *In vivo* biocompatibility evaluation of HSDF-NH_2_

On day 14 post-administration, comprehensive blood panel analyses and serum biochemistry tests were performed using collected blood samples. These tests included measurements of aspartate aminotransferase (AST) and alanine aminotransferase (ALT). Additionally, major organs—such as the heart, liver, spleen, lungs, and kidneys—were harvested from the rats for subsequent histological analysis.

### Enzyme-linked immunosorbent assay (ELISA)

To assess the expression of inflammation-related cytokines in the ischemic hemisphere, brain tissues were promptly collected and homogenized in cold PBS. Following centrifugation at 12000g for 15 min at 4°C, the content of TNF-α and IL-1β in the samples were quantified using an ELISA kit following standard protocols.

### Statistical analysis

All results were reported as means ± standard deviation (SD). Significance was determined by the student's t-test or one-way analysis of variance (ANOVA) using GraphPad Prism (version 8). The statistical significance was considered when the *P* value was less than 0.05.

## Supplementary Material

Supplementary methods, figures and table.

## Abbreviations

H_2_S: hydrogen sulfide
BBB: blood-brain barrier
ROS: reactive oxygen species
I/R: ischemia-reperfusion
rt-PA: recombinant tissue plasminogen activator
SOD: superoxide dismutase
GSH-PX: glutathione peroxidase
MDA: malondialdehyde
LR: Lawesson's Reagent
COS: carbonyl sulfide
CA: carbonic anhydrase
OGD/R: oxygen-glucose deprivation/reoxygenation
FITC: fluorescein isothiocyanate
PI: propidium iodide
tMCAO/R: transient middle cerebral artery occlusion/reperfusion
TTC: 2,3,5-Triphenyltetrazolium chloride
AST: aspartate aminotransferase
ALT: alanine aminotransferase
ELISA: Enzyme-linked immunosorbent assay
HRMS: high-resolution mass spectrometry
DHE: dihydroethidium
ECA: External carotid artery
ICA: Internal carotid artery
PPA: Paramedian pontine arteries
PCA: Posterior cerebral artery
MCA: Middle cerebral artery
ACA: Anterior cerebral artery
logD: oil-water distribution coefficient
