# Supplementary material for: A fluorogenic ROS-triggered hydrogen sulfide donor for alleviating cerebral ischemia-reperfusion injury
Source: Theranostics. 2024 Nov 4;14(19):7589–603. doi: 10.7150/thno.100930 (PMC11626942; doi:10.7150/thno.100930)
Supplement: Supplementary file 1 — Supplementary methods, figures and table. [file thnov14p7589s1.pdf]

Supplementary Data for

A fluorogenic ROS-triggered hydrogen sulfide donor for alleviating cerebral ischemia-reperfusion injury

*Huangjie Lu[#, a, b], Huiying Zeng[#, a, b], Wenlong Wei[#, c], Yuying Chen[a, b],Ziqiang Zhou[a, b], Xuyang Ning[a, b], Ping Hu [a, b]**

[a] *State Key Laboratory of Bioactive Molecules and Druggability Assessment, Jinan University, Guangzhou 510006, China.*

[b] *College of Pharmacy, Jinan University, Guangzhou 510006, China.*

[C] *Department of Burns & Plastic Surgery, Guangzhou Red Cross Hospital, Faculty of Medical Science, Jinan University, Guangzhou 510006, China*

[#]: These authors contributed equally.

Corresponding author: Ping Hu*

Mailing address: Jinan University, No.855, East Xingye Avenue, Panyu Districst, Guangzhou 510632, CHINA

Tel.: +86-18581483142

E-mail: inzahu@hotmail.com

S1. Synthesis of Compound 1-7, HSDF-NH_2, CODF-NH_2 and HSDG-NH_2

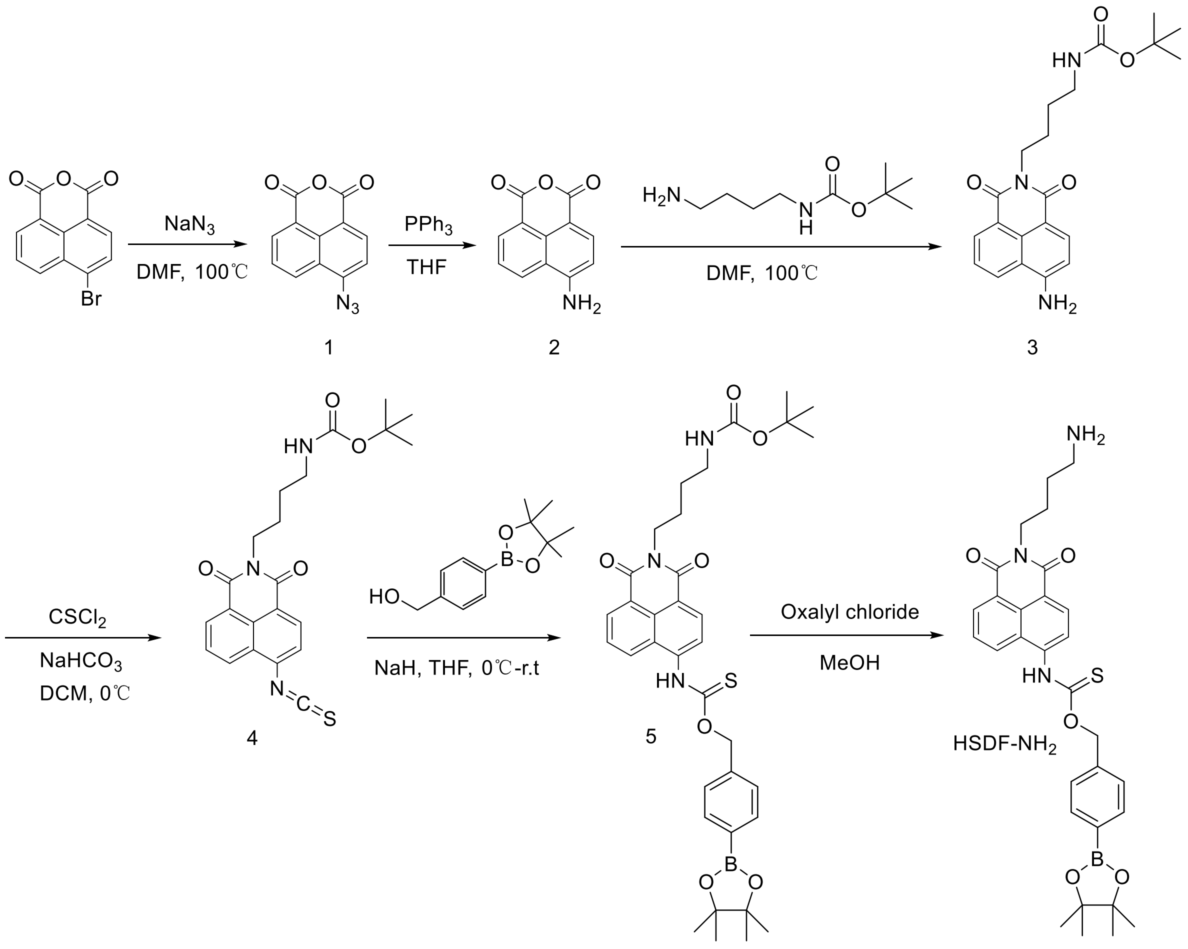

Scheme S1. Synthesis of HSDF-NH_2

Synthetic procedures and characterizations:

Synthesis of compound 1

Add 4-Bromo-1,8-naphthalic anhydride (13 g, 46.9 mmol) to 70 mL DMF, stir for 30 min until dissolved. Dissolve sodium azide (3.5 g, 53.8 mmol) in 3 mL of water and add to the DMF solution. The mixture was heated to 100°C and the reaction was stopped after 15 min. Add water and collect the precipitate by filtration, wash with water and dry in vacuum to obtain compound 1. Yield: 10.09g, 90%. ^{1}H NMR (400 MHz, Chloroform-*d*) δ 8.70 – 8.52 (m, 3H), 7.86 – 7.77 (m, 1H), 7.54 (d, J = 8.0 Hz, 1H). ^{13}C NMR (101 MHz, Chloroform-*d*) δ 160.47, 159.93, 145.20, 134.37, 133.94, 131.37, 130.33, 127.32, 124.51, 118.81, 115.12, 114.60.

Synthesis of compound 2

Compound 1 (3.68 g, 15.385 mmol) was suspended in a mixture of 30 mL THF and 60 mL 0.5 M HCl, and triphenylphosphine (5.25 g, 20 mmol) was slowly added to the suspension under stirring. After stirring for an additional 30 min, the mixture was basified with 20 mL of 2 M aqueous NaOH and the THF was removed by rotary evaporation. The resulting mixture was diluted with ethyl acetate and collect the precipitate by filtration. The product was washed with ethyl acetate and water, and dried under vacuum to obtain compound 2. Yield: 2.62g, 80%. ^{1}H NMR (400 MHz, DMSO-d_6) δ 8.62 (d, J = 8.4 Hz, 1H), 8.36 (d, J = 7.2 Hz, 1H), 8.12 (d, J = 8.5 Hz, 1H), 7.73 (s, 2H), 7.62 (t, J = 7.9 Hz, 1H), 6.82 (d, J = 8.5 Hz, 1H). ^{13}C NMR (101 MHz, DMSO-d_6) δ 162.42, 160.73, 154.33, 136.25, 133.37, 132.94, 131.09, 124.72, 119.70, 118.58, 109.16, 102.63.

Synthesis of compound 3

Compound 2 (639.57 mg, 3 mmol) and tert-Butyl N-(4-aminobutyl)carbamate (1.129 g, 6 mmol) were heated to 100°C in 15 mL DMF overnight. Add cold water and collect the precipitate by filtration. The residue was purified by flash column chromatography to obtain compound 3. Yield: 804mg, 70%. ^{1}H NMR (400 MHz, DMSO-d_6) δ 8.60 (d, J = 8.4 Hz, 1H), 8.41 (d, J = 7.2 Hz, 1H), 8.18 (d, J = 8.4 Hz, 1H), 7.63 (t, J = 7.8 Hz, 1H), 7.42 (s, 2H), 6.84 (d, J = 8.4 Hz, 1H), 6.77 (t, J = 5.8 Hz, 1H), 3.99 (t, J = 7.1 Hz, 2H), 2.98 – 2.86 (m, 2H), 1.64 – 1.53 (m, 2H), 1.47 – 1.37 (m, 2H), 1.35 (s, 9H). ^{13}C NMR (101 MHz, DMSO-d_6) δ 164.24, 163.37, 156.03, 153.14, 134.38, 131.42, 130.14, 129.72, 124.40, 122.24, 119.83, 108.62, 108.03, 77.79, 28.71, 27.70, 25.78.

Synthesis of compound 4

To an anhydrous DCM solution of compound 3 (1.43 g, 3.73 mmol) and $NaHCO_3$ (630 mg, 7.50 mmol), thiophosgene (0.858 mg, 7.45 mmol) in anhydrous DCM was added dropwise over 30 min at 0°C. The reaction mixture was stirred overnight at r.t. After removing the solvent, the residue was purified by silica-gelchromatography to obtain compound 4. Yield: 951 mg, 60%. ^{1}H NMR (400 MHz, Chloroform-*d*) δ 8.64 (dd, J = 7.3, 1.1 Hz, 1H), 8.52 (d, J = 7.9 Hz, 1H), 8.45

(dd, J = 8.4, 1.1 Hz, 1H), 7.85 (dd, J = 8.4, 7.4 Hz, 1H), 7.63 (d, J = 7.9 Hz, 1H), 4.67 (s, 1H), 4.18 (t, J = 7.4 Hz, 2H), 3.21 (q, J = 6.7 Hz, 2H), 1.82 – 1.73 (m, 2H), 1.68 – 1.58 (m, 2H), 1.44 (s, 9H). ^{13}C NMR (101 MHz, Chloroform-*d*) δ 163.63, 163.12, 155.93, 133.96, 132.17, 131.26, 128.88 (d, J = 8.1 Hz), 127.90, 127.48, 124.44, 123.11, 121.02, 40.05, 28.42, 27.59, 25.41. MS(ESI) m/z: 426.14 $[M+H]^+$.

Synthesis of compound 5

NaH (37 mg, 1.61 mmol, 60% in paraffin liquid) was dissolved in 15 mL anhydrous THF, phenylboronic acid pinacol ester (377 mg, 1.61 mmol) was added to this solvent at 0°C. Then compound 4 (684 mg, 1.61 mmol) was dissolved in 10 mL THF and added to the mixture dropwise. The resultant mixture was stirred at 0°C for 30 min, then stirred at r.t. overnight. The reaction was quenched by adding brine and the reactant was extracted with ethyl acetate. The organic layers were combined and dried over $MgSO_4$, filtered and evaporated under reduced pressure to obtain the crude product, which was purified by silica gel flash chromatography to obtain compound 5. Yield: 160 mg, 15%. ^{1}H NMR (400 MHz, Chloroform-*d*) δ 9.29 (s, 1H), 8.54 (dd, J = 20.2, 7.6 Hz, 2H), 8.30 (d, J = 8.5 Hz, 1H), 7.91 (s, 1H), 7.74 (dd, J = 24.6, 7.8 Hz, 3H), 7.38 – 7.26 (m, 2H), 5.61 (s, 2H), 4.71 (s, 1H), 4.14 (t, J = 7.4 Hz, 2H), 3.16 (q, J = 6.8 Hz, 2H), 1.79 – 1.68 (m, 2H), 1.61 – 1.54 (m, 2H), 1.41 (s, 9H), 1.35 (s, 12H). ^{13}C NMR (101 MHz, Chloroform-*d*) δ 189.47, 163.95, 163.45, 137.66, 135.04, 131.58, 131.39, 128.90, 128.31, 127.47, 127.18, 122.99, 120.65, 83.97, 73.48, 39.91, 28.41, 27.52, 25.39, 24.87. MS(ESI) m/z: 660.28 $[M+H]^+$.

Synthesis of HSDF-NH_2

Compound 5 (131.8 mg, 0.2 mmol) was dissolved in methanol, then oxalyl chloride (51.18 μl, 0.6 mmol) was added dropwise to the above solution, and stirred at room temperature for 1 hour. After the reaction was completed, the crude product was obtained by evaporation under reduced pressure. The crude material was then extracted with dichloromethane and washed with brine. The organic layer was dried over anhydrous $MgSO_4$ and filtered. After concentration, the final product was purified by silica gel flash chromatography. Yield: 33.54 mg, 30%. ^{1}H NMR

(400 MHz, DMSO-d_6) δ 8.51 (dd, J = 10.0, 7.5 Hz, 2H), 8.38 (dd, J = 8.5, 1.1 Hz, 1H), 7.93 – 7.87 (m, 2H), 7.68 (d, J = 7.5 Hz, 2H), 7.42 (d, J = 7.5 Hz, 2H), 5.58 (s, 2H), 4.14 (s, 2H), 4.08 (t, J = 6.7 Hz, 2H), 2.82 (t, J = 7.4 Hz, 2H), 1.71 (p, J = 6.7 Hz, 2H), 1.61 (q, J = 8.4, 7.6 Hz, 2H), 1.29 (s, 12H). ^{13}C NMR (151 MHz, DMSO-d_6) δ 163.94, 163.47, 158.43 (q, J = 31.4 Hz), 139.49, 134.99, 131.56, 131.32, 130.35, 128.76, 127.71, 127.49, 122.84, 120.62, 118.63, 116.65, 114.66, 84.19, 73.99, 71.79, 49.05, 39.09, 25.41, 25.13 (d, J = 3.5 Hz). HRMS(ESI) m/z: $[M+H]^+$, calcd for $C30H35BN3O5S^+$: 560.2312, found: 560.2383.

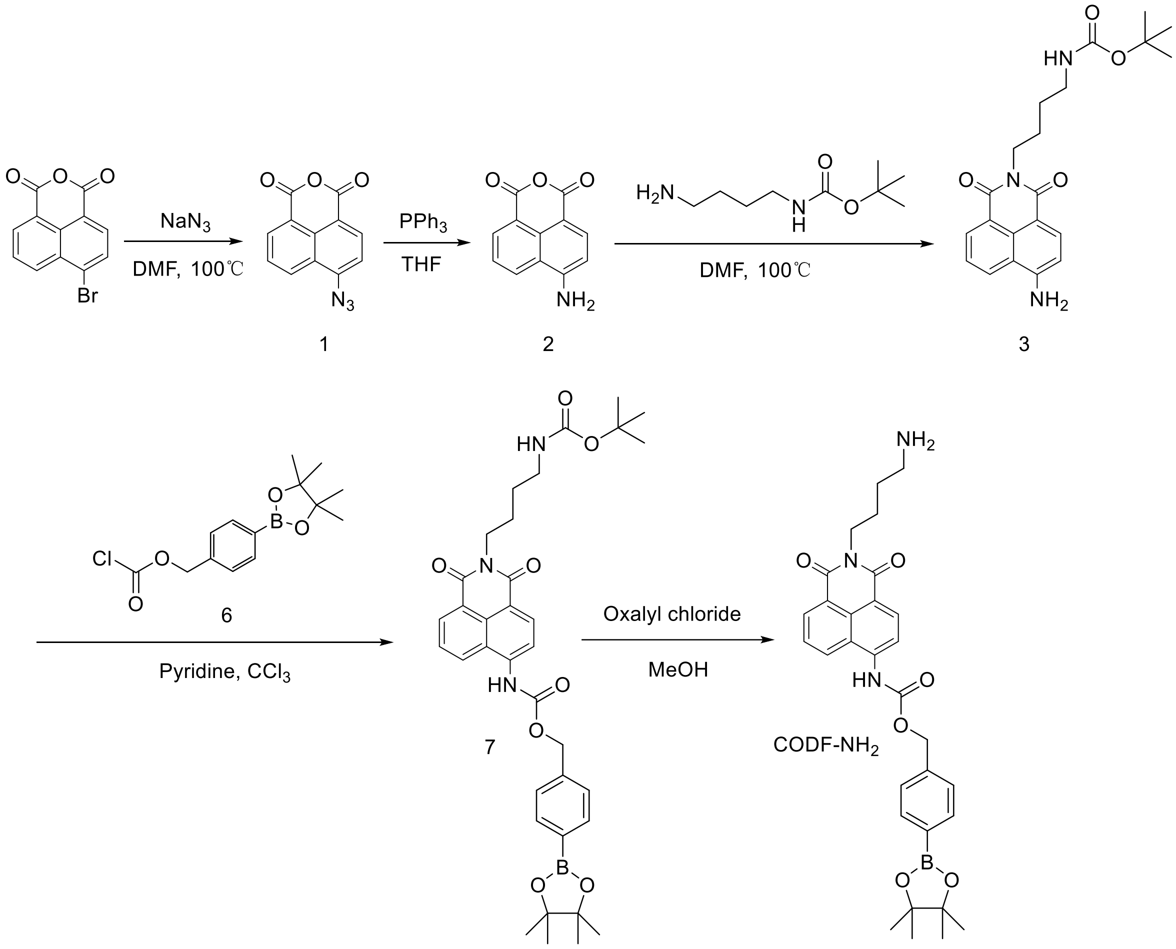

Scheme S2. Synthesis of CODF-NH_2

Synthetic procedures and characterizations:

Synthesis of compound 6

Place Na_2CO_3 (7 g, 66 mmol) into a dry round-bottomed flask and add triphosgene (4.4 g, 14.8 mmol) in 30 mL of toluene at 0°C. Dissolve (4-(4,4,5,5-Tetramethyl-1,3,2-dioxaborolan-2-

yl)phenyl)methanol (1.74 g, 7.4 mmol) in 10 mL toluene, add it to the above system at 0°C and stir for 1 hour. After stirring at r.t. for 6 hours, the mixture was filtered through a Celite pad. The solvent was removed in vacuo without further purification. Yield: 658.4 mg, 30%.

Synthesis of compound 7

Compound 3 (2.276 g, 5.94 mmol) was dissolved in 30 mL of anhydrous THF, and Na_2CO_3 (3.5 g, 33 mmol) was added. Compound 6 (1.8 g, 6.069 mmol) was dissolved in 60 mL DCM solution and added dropwise to the above solution. After stirring at room temperature overnight, the solvent was removed in vacuo to obtain the crude product, which was purified by silica gel flash chromatography to obtain compound 7. Yield: 649.61 mg, 17%. ^{1}H NMR (400 MHz, Chloroform-*d*) δ 8.55 (dd, J = 12.0, 7.7 Hz, 2H), 8.32 (d, J = 8.3 Hz, 1H), 8.23 (d, J = 8.5 Hz, 1H), 7.93 (s, 1H), 7.84 (d, J = 7.9 Hz, 2H), 7.70 (dd, J = 8.5, 7.3 Hz, 1H), 7.43 (d, J = 7.7 Hz, 2H), 5.30 (s, 2H), 4.72 (s, 1H), 4.16 (t, J = 7.4 Hz, 2H), 3.18 (q, J = 6.5 Hz, 2H), 1.80 – 1.71 (m, 2H), 1.63 – 1.55 (m, 2H), 1.43 (s, 9H), 1.36 (s, 12H). ^{13}C NMR (101 MHz, Chloroform-*d*) δ 164.13, 163.62, 156.04, 153.26, 144.12, 139.25, 138.35, 135.13, 135.00, 132.45, 131.25, 127.61, 126.49, 126.05, 123.13, 83.98, 83.80, 67.73, 65.15, 40.22, 39.84, 28.42, 27.53, 25.42, 24.86. MS(ESI) m/z: 644.30 $[M+H]^+$.

Synthesis of CODF-NH_2

Compound 7 (192 mg, 0.298 mmol) was dissolved in methanol, then oxalyl chloride (76.37 μl, 0.9 mmol) was added dropwise to the above solution, and stirred at room temperature for 1 hour. After the reaction was completed, the crude product was obtained by evaporation under reduced pressure. The crude material was then extracted with dichloromethane and washed with brine. The organic layer was dried over anhydrous $MgSO_4$ and filtered. After concentration, the final product was purified by silica gel flash chromatography. Yield: 48.5 mg, 30%. ^{1}H NMR (400 MHz, DMSO-d_6) δ 8.75 – 8.69 (m, 1H), 8.53 – 8.43 (m, 2H), 8.19 (d, J = 8.2 Hz, 1H), 7.83 (dd, J = 8.6, 7.3 Hz, 1H), 7.75 – 7.69 (m, 2H), 7.51 (d, J = 7.7 Hz, 2H), 5.31 (s, 2H), 4.06 (t, J = 6.7 Hz, 2H), 3.36 (s, 2H), 2.80 (t, J = 7.3 Hz, 2H), 1.77 – 1.66 (m, 2H), 1.66 – 1.55 (m, 2H), 1.30 (s, 12H).

^{13}C NMR (101 MHz, DMSO-d_6) δ 164.01, 163.45, 154.37, 141.22, 140.07, 135.07, 132.21, 131.44, 129.89, 128.79, 127.73, 126.86, 124.31, 122.59, 118.68, 117.47, 84.19, 66.74, 49.05, 39.02, 25.43, 25.14. HRMS(ESI) m/z: $[M+H]^+$, calcd for $C30H35BN3O6^+$: 544.2541, found: 544.2609.

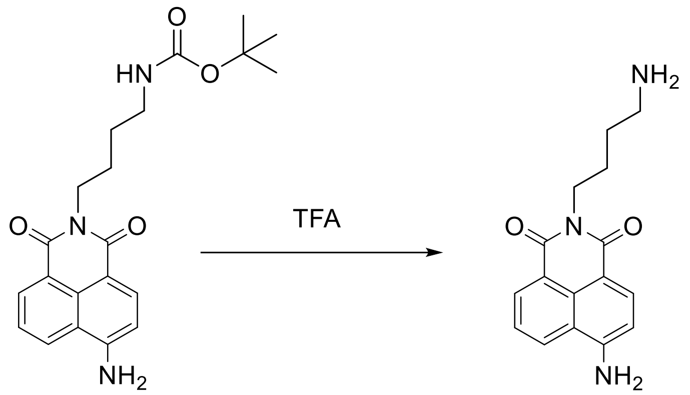

3 HSDG-NH_2

Scheme S3. Synthesis of HSDG-NH_2

Synthetic procedures and characterizations:

Synthesis of HSDG-NH_2

Compound 3 (383.18 mg, 1 mmol) was dissolved in methanol, then oxalyl chloride (225.9 µl, 3 mmol) was added dropwise to the above solution, and stirred at room temperature for 1 hour. After the reaction was completed, the crude product was obtained by evaporation under reduced pressure. The crude material was then extracted with dichloromethane and washed with brine. The organic layer was dried over anhydrous $MgSO_4$ and filtered. After concentration, the final product was purified by silica gel flash chromatography. Yield: 90.6 mg, 32%. ^{1}H NMR (400 MHz, DMSO-d_6) δ 8.70 (d, J = 8.4 Hz, 1H), 8.42 (d, J = 7.2 Hz, 1H), 8.18 (d, J = 8.4 Hz, 1H), 8.00 (s, 2H), 7.64 (t, J = 7.8 Hz, 1H), 7.61 (s, 2H), 6.87 (d, J = 8.4 Hz, 1H), 4.03 (t, J = 6.6 Hz, 2H), 2.77 (t, J = 7.2 Hz, 2H), 1.71 – 1.56 (m, 4H). ^{13}C NMR (101 MHz, DMSO-d_6) δ 164.36, 163.45, 159.11, 158.80, 153.36, 134.47, 131.51, 129.92, 124.39, 119.07, 108.65, 49.02, 25.33, 25.16. HRMS(ESI) m/z: $[M+H]^+$, calcd for $C16H18N3O2^+$: 284.1321, found: 284.1390.

S2. Spectroscopic Analysis

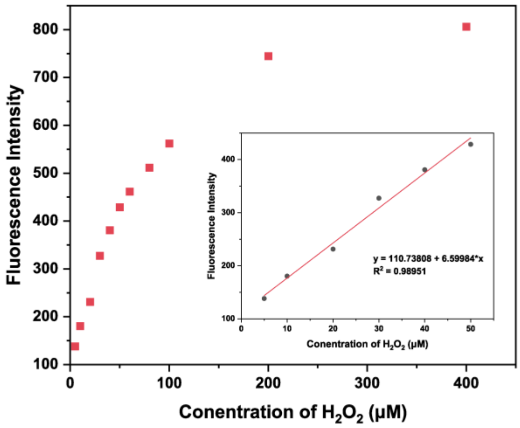

Figure S1. Fluorescence intensity changes with various concentration of H_2O_2 recorded after 40 min of reaction, inset: the linear fitting curve in the range of 0–50 μM H_2O_2.

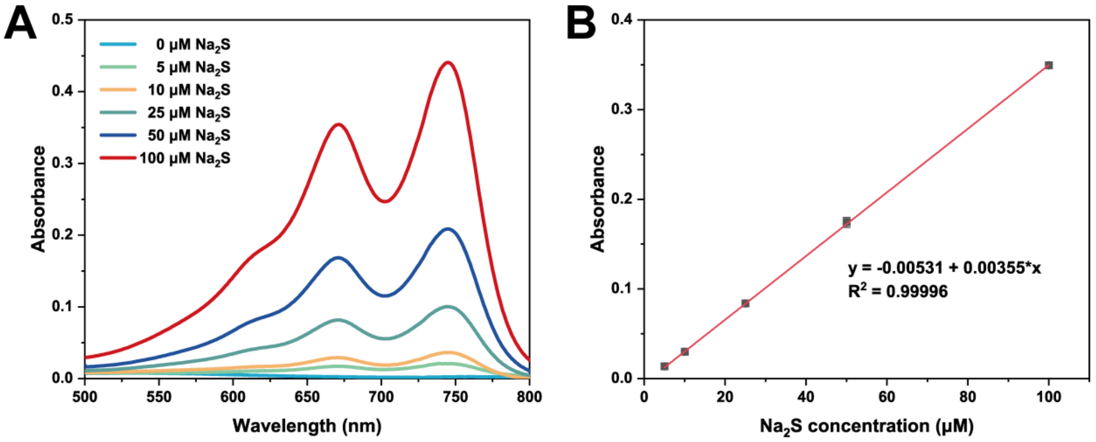

Figure S2. (A) UV-Vis absorption spectrum of Na_2S at different concentrations via MB assay. (B) H_2S calibration curve obtained with Na_2S.

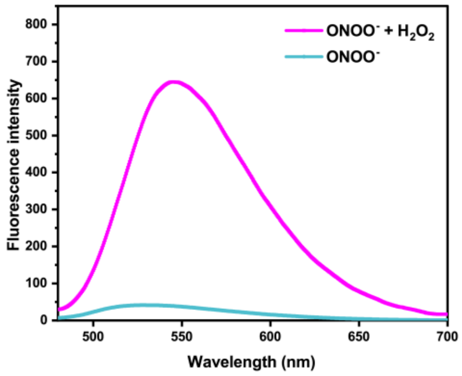

Figure S3. Fluorescence intensity of 10μM HSDF-NH_2 reacting first with ONOO- (100 μM) and then with H_2O_2 (100 μM).

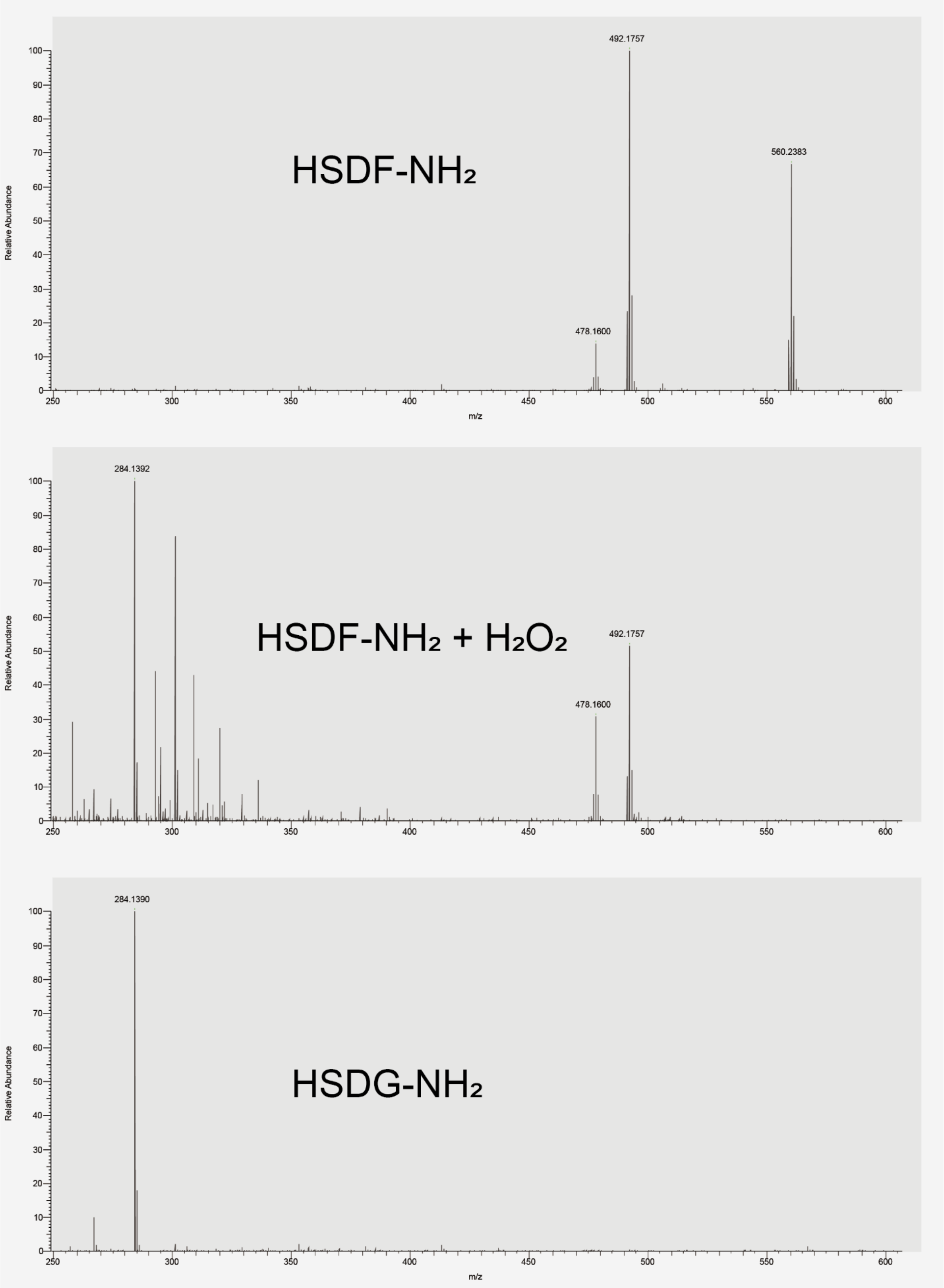

Figure S4. HRMS traces of the samples: HSDF-NH_2, HSDF-NH_2 after reacting with H_2O_2 in PBS buffer for 120 min, and HSDG-NH_2.

S3. Cells Assay

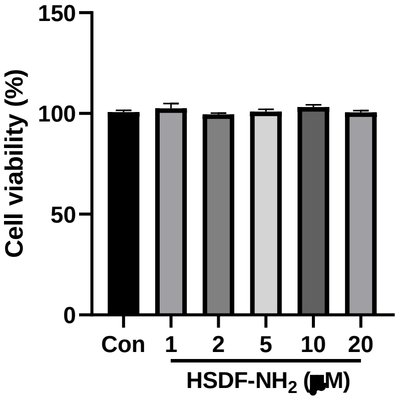

Figure S5. *In vitro* cytotoxicity evaluation of $HSDF\text{-}NH_2$ in PC-12 cells after incubation for 24 h. Data are expressed as means ± SD (n = 5).

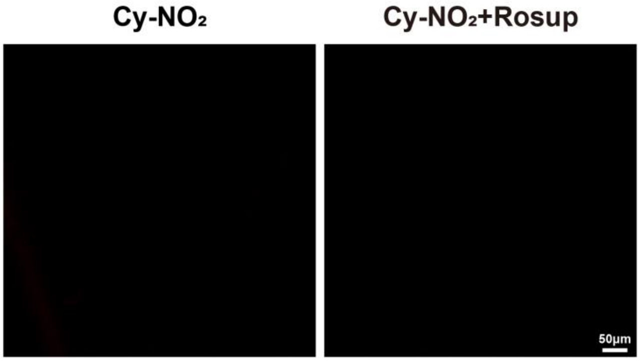

Figure S6. Confocal microscopy images of PC-12 cells treated with Rusup in $Cy\text{-}NO_2$ channel in the absence of $HSDF\text{-}NH_2$.

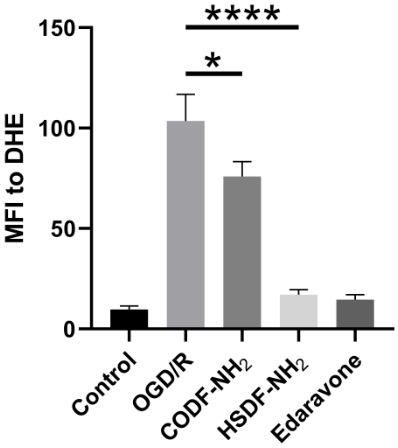

Figure S7. The quantified results of fluorescence in Figure 3B. Data are expressed as means ± SD (n = 3).

S4. Biological Data

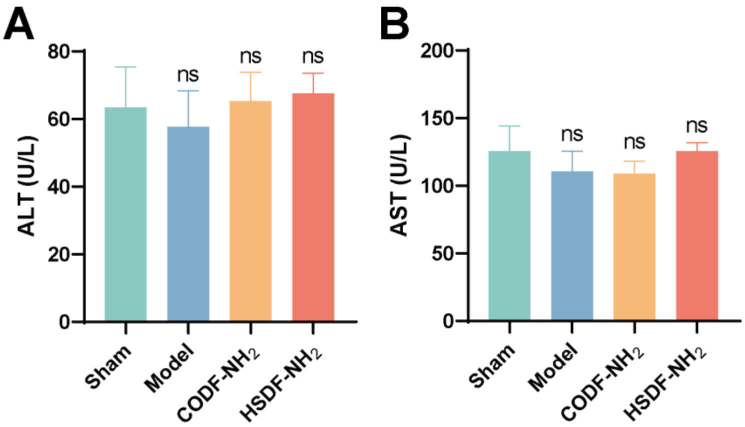

Figure S8. (A) The level of (a) ALT and (b) AST in rats after treated with different drugs. Data are expressed as means ± SD (n = 3).

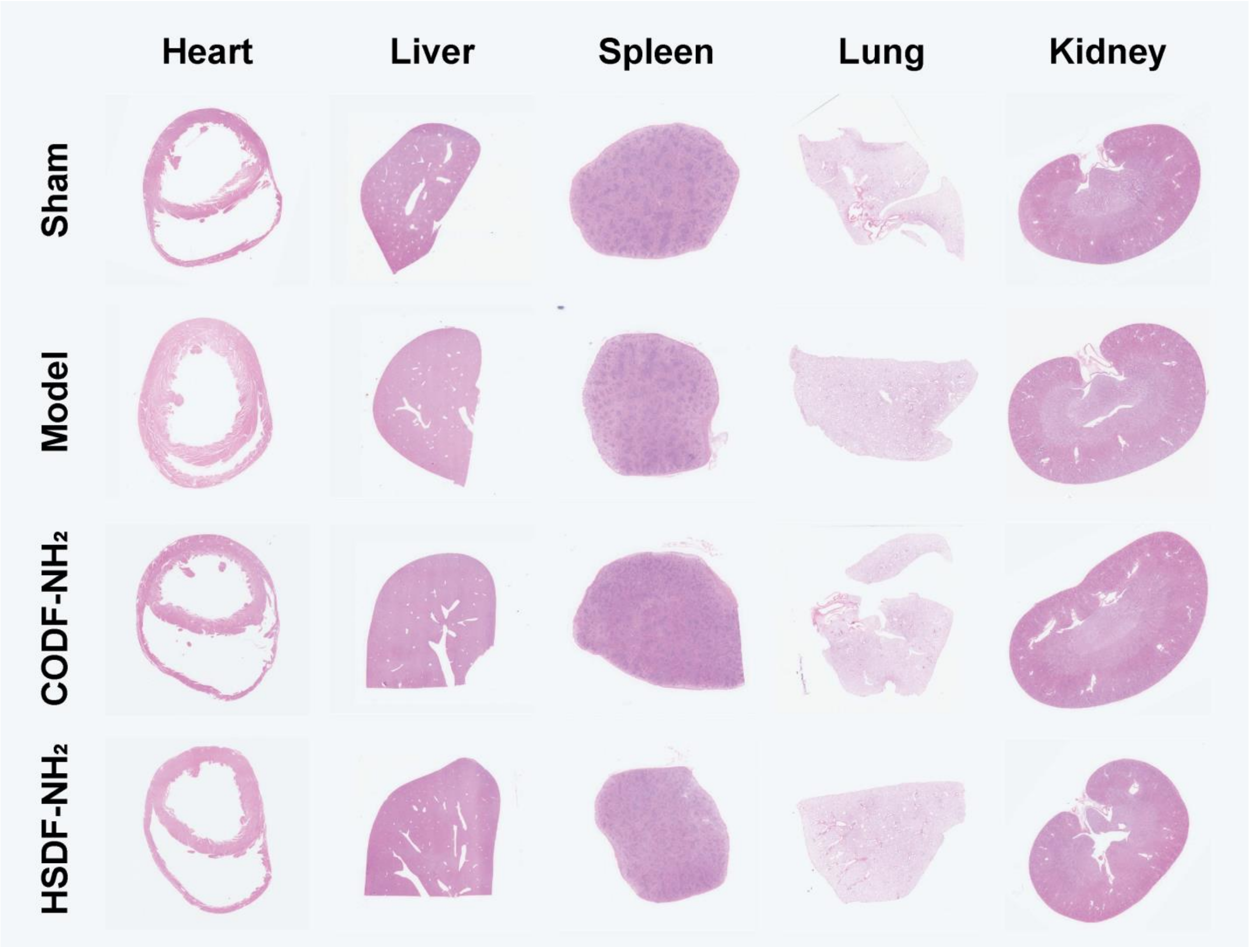

Figure S9. The H&E staining images of main organs with full view.

S5. NMR Spectra and HRMS Spcetrum

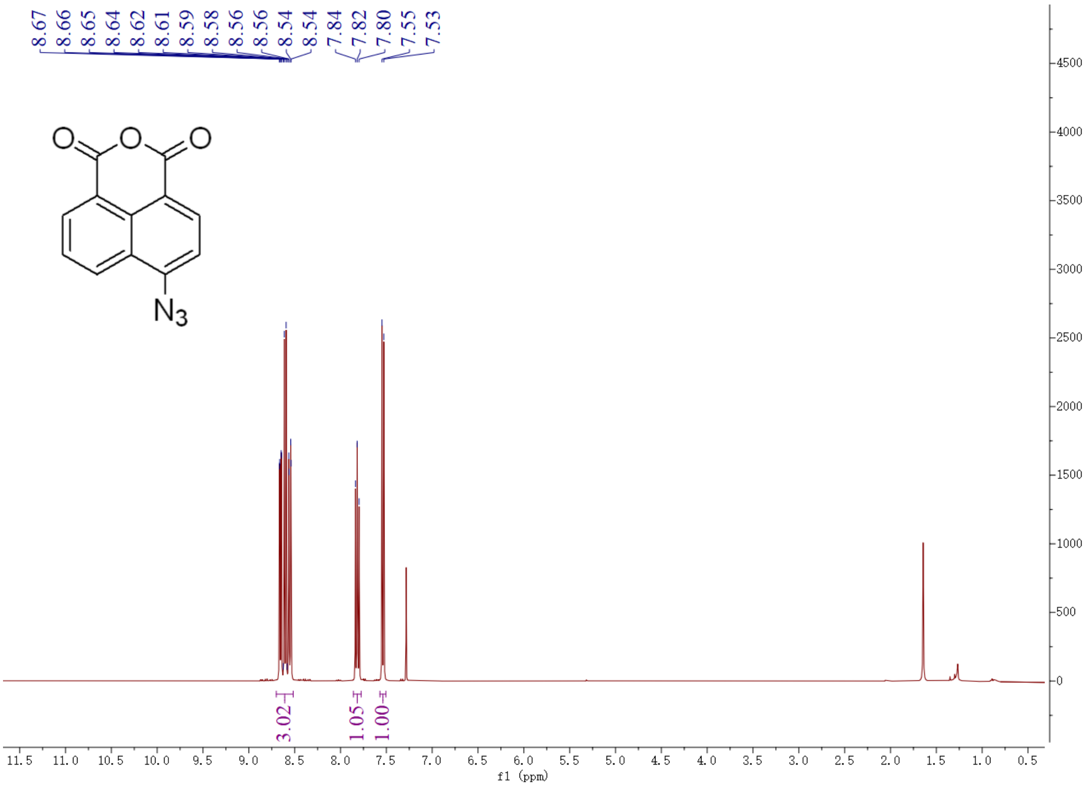

Figure S10. ^{1}H NMR spectrum (400 MHz, Chloroform-d) of compound **1**.

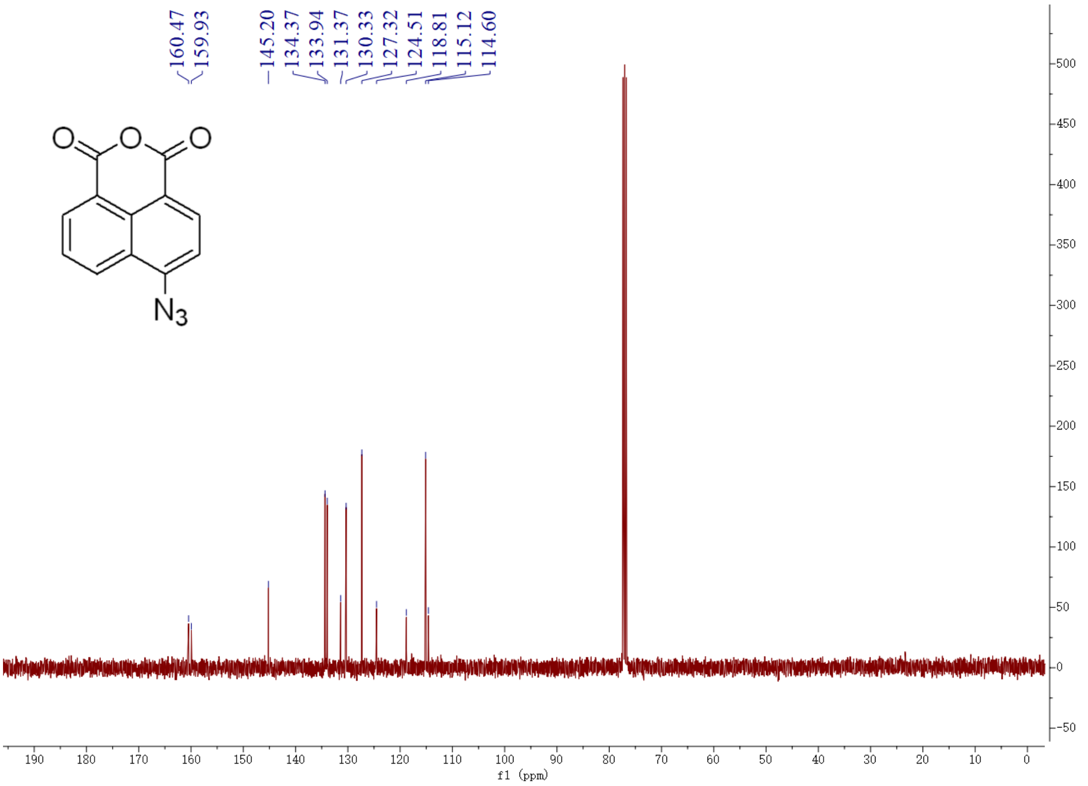

Figure S11. ^{13}C NMR spectrum (101 MHz, Chloroform-d) of compound **1**.

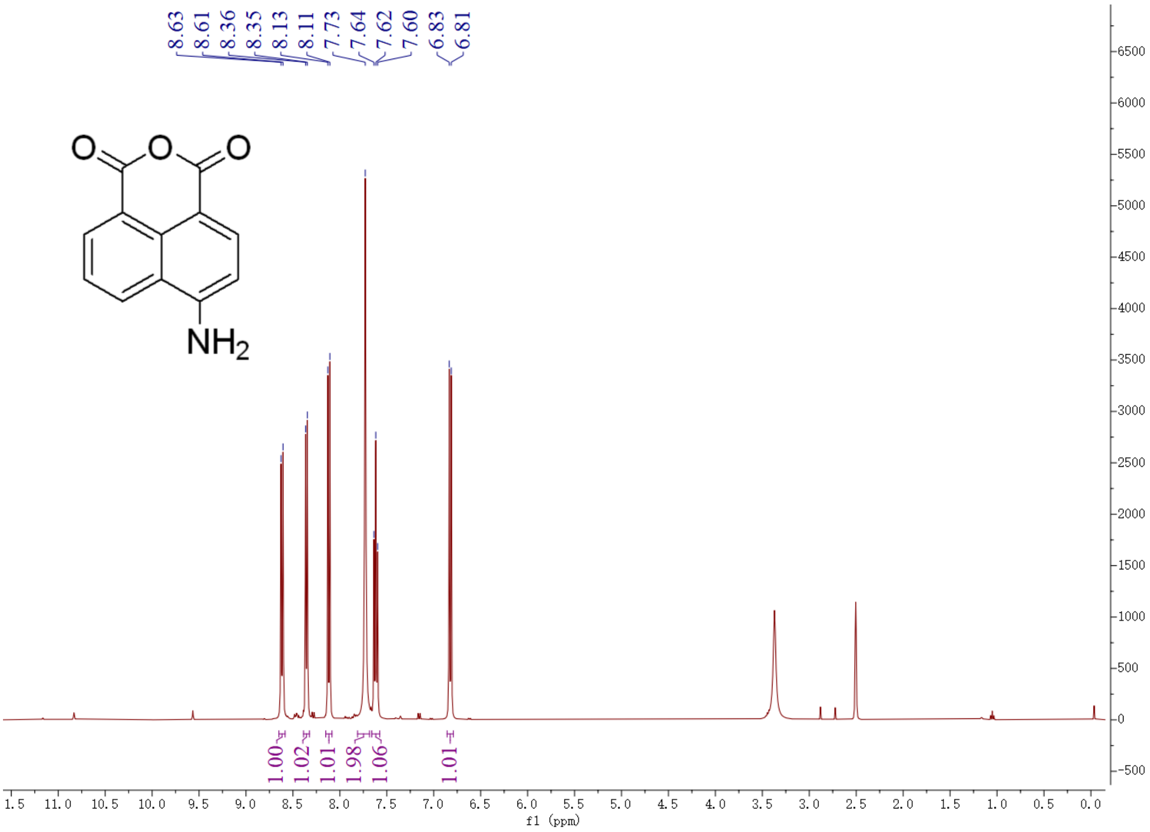

Figure S12. ^{1}H NMR spectrum (400 MHz, DMSO-d_6) of compound **2**.

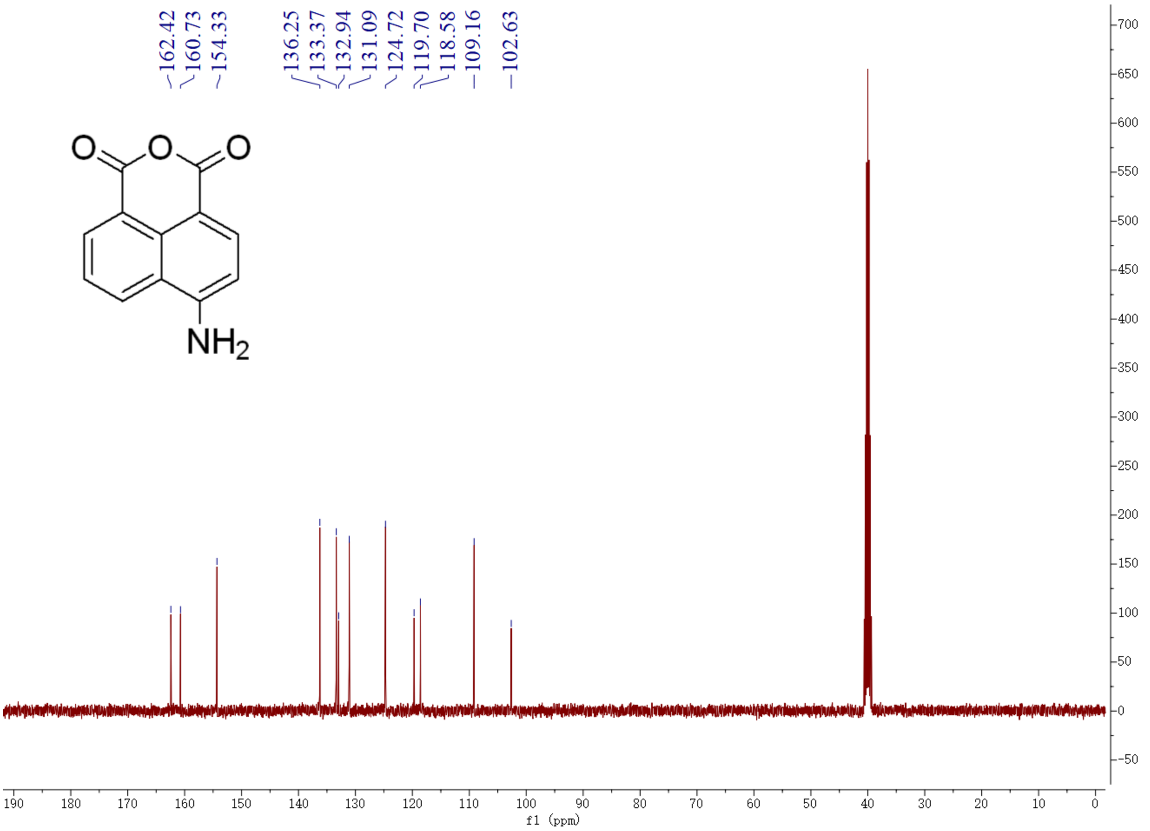

Figure S13. ^{13}C NMR spectrum (101 MHz, DMSO-d_6) of compound **2**.

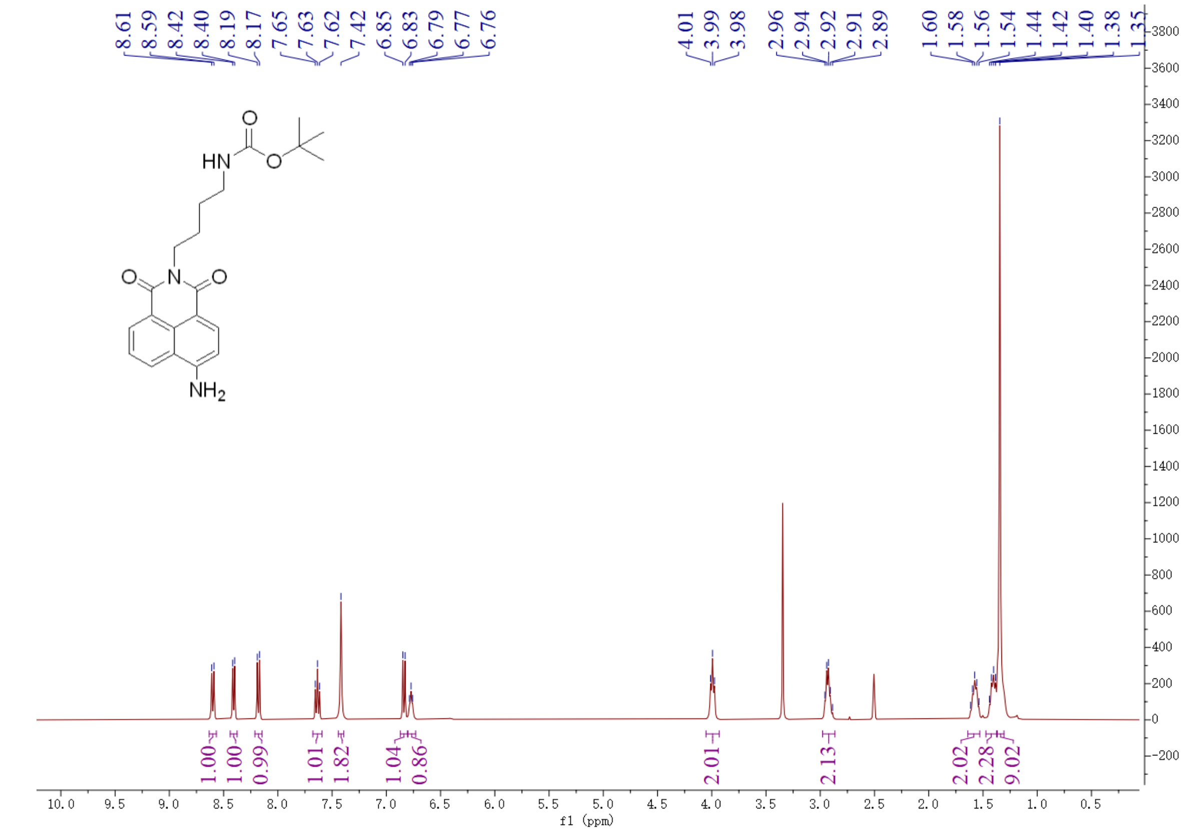

Figure S14. ^{1}H NMR spectrum (400 MHz, DMSO-d_6) of compound **3**.

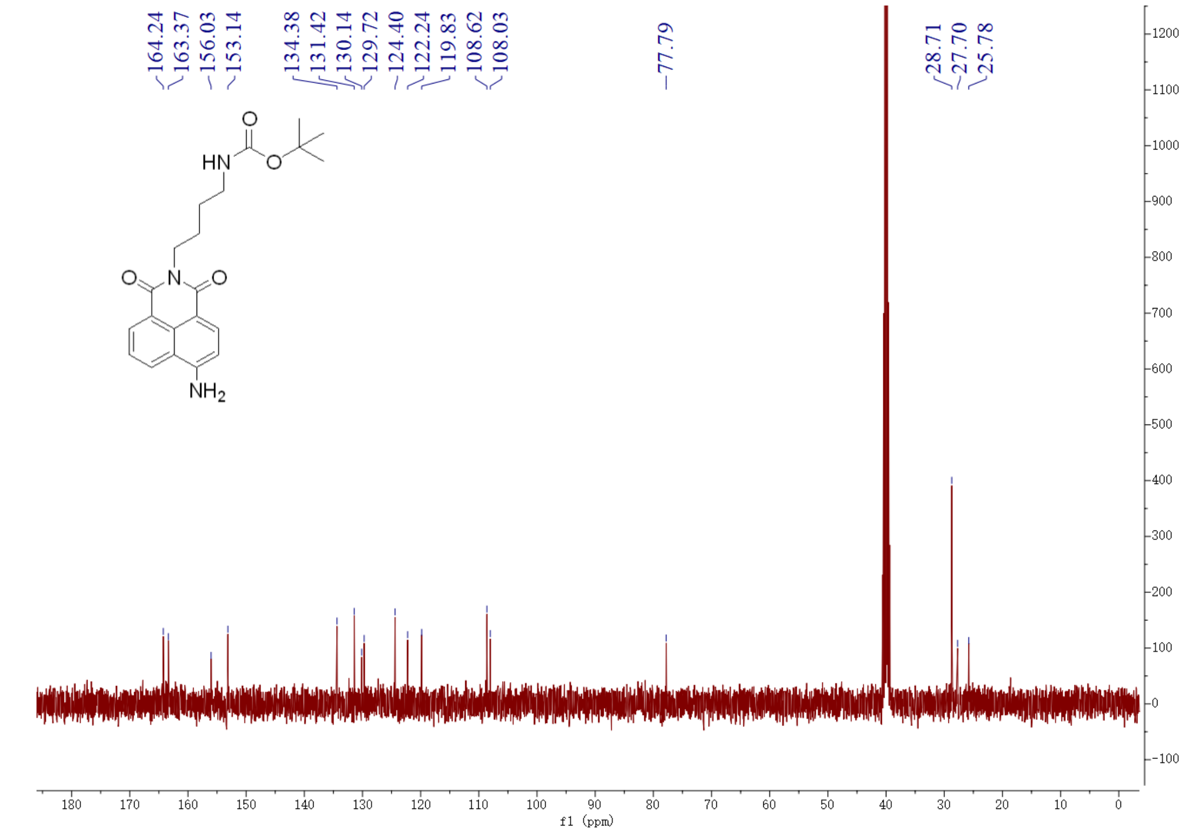

Figure S15. ^{13}C NMR spectrum (101 MHz, DMSO-d_6) of compound **3**.

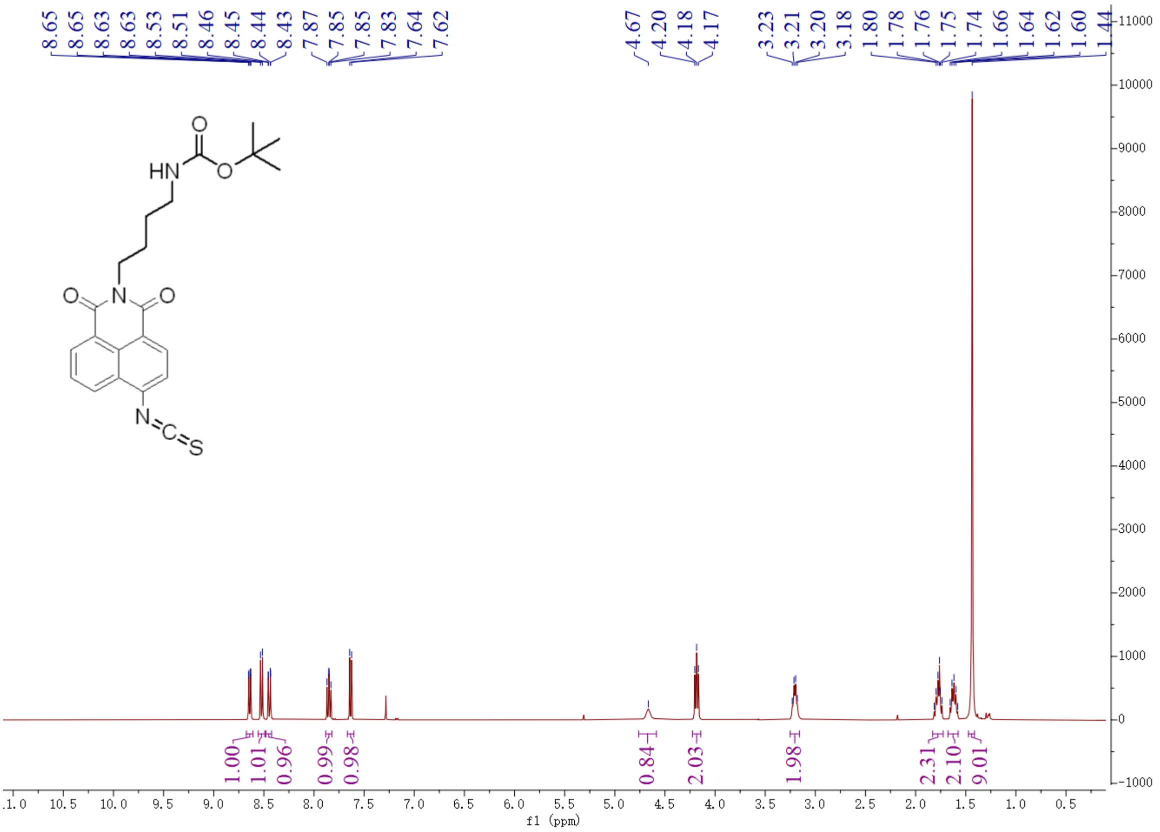

Figure S16. ^{1}H NMR spectrum (400 MHz, Chloroform-d) of compound **4**.

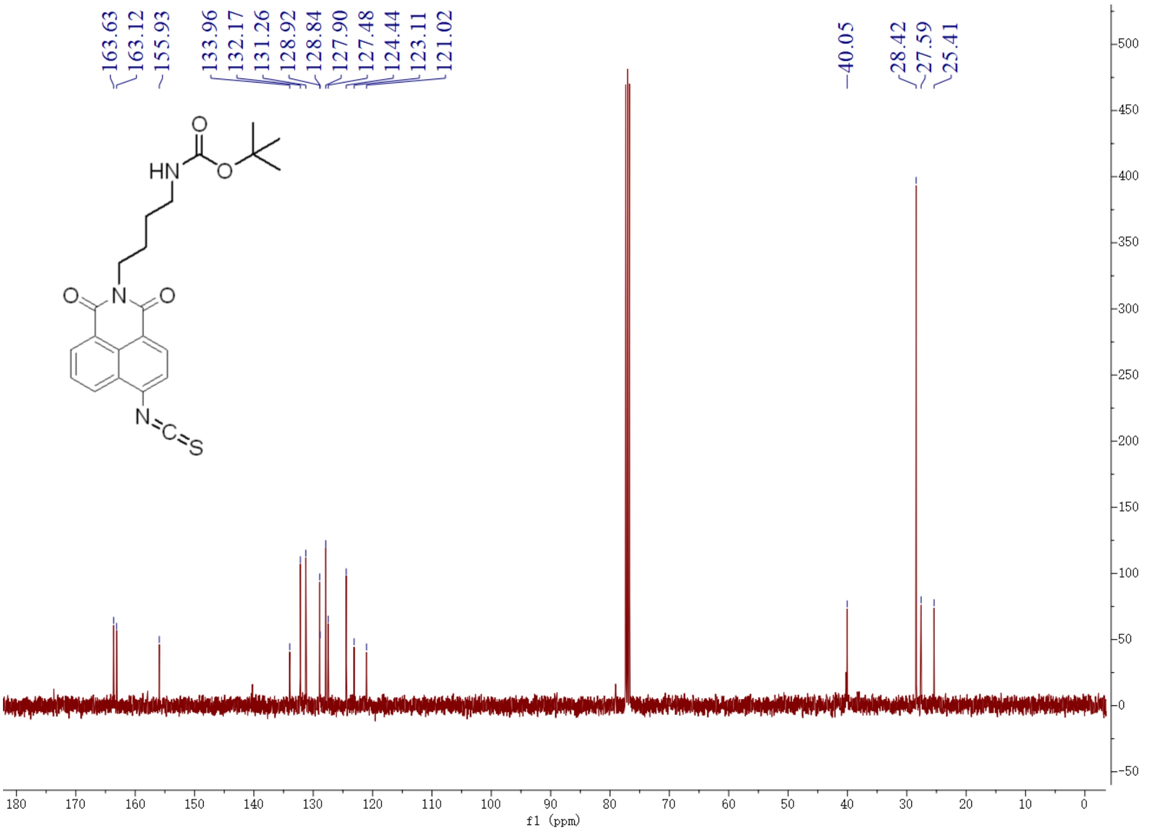

Figure S17. ^{13}C NMR spectrum (101 MHz, Chloroform-d) of compound **4**.

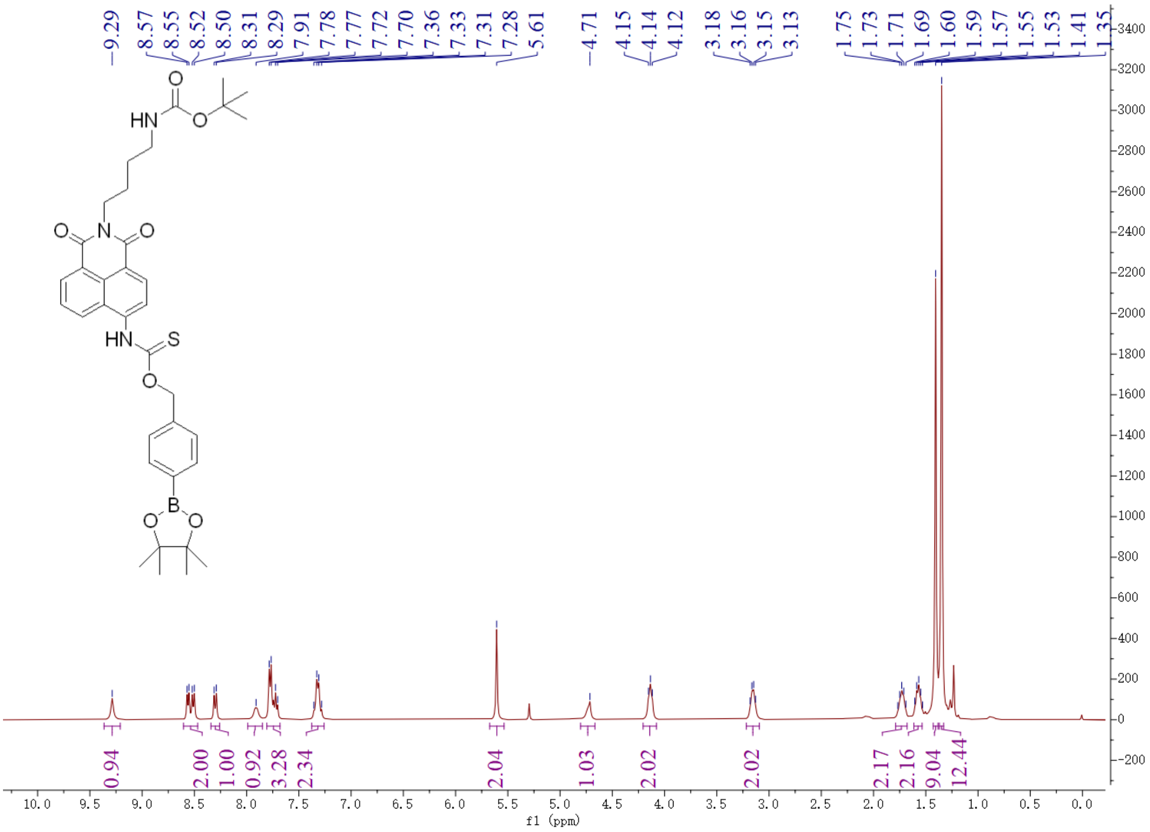

Figure S18. ^{1}H NMR spectrum (400 MHz, Chloroform-d) of compound **5**.

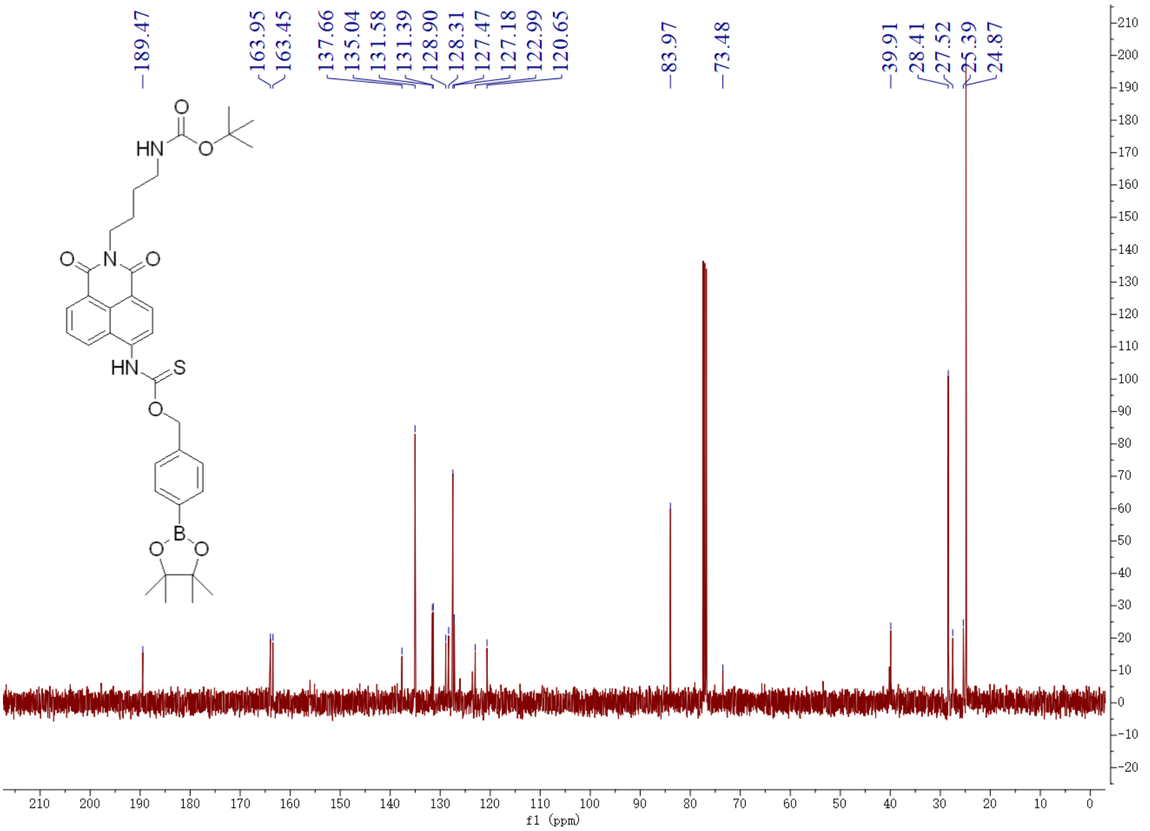

Figure S19. ^{13}C NMR spectrum (101 MHz, Chloroform-d) of compound **5**.

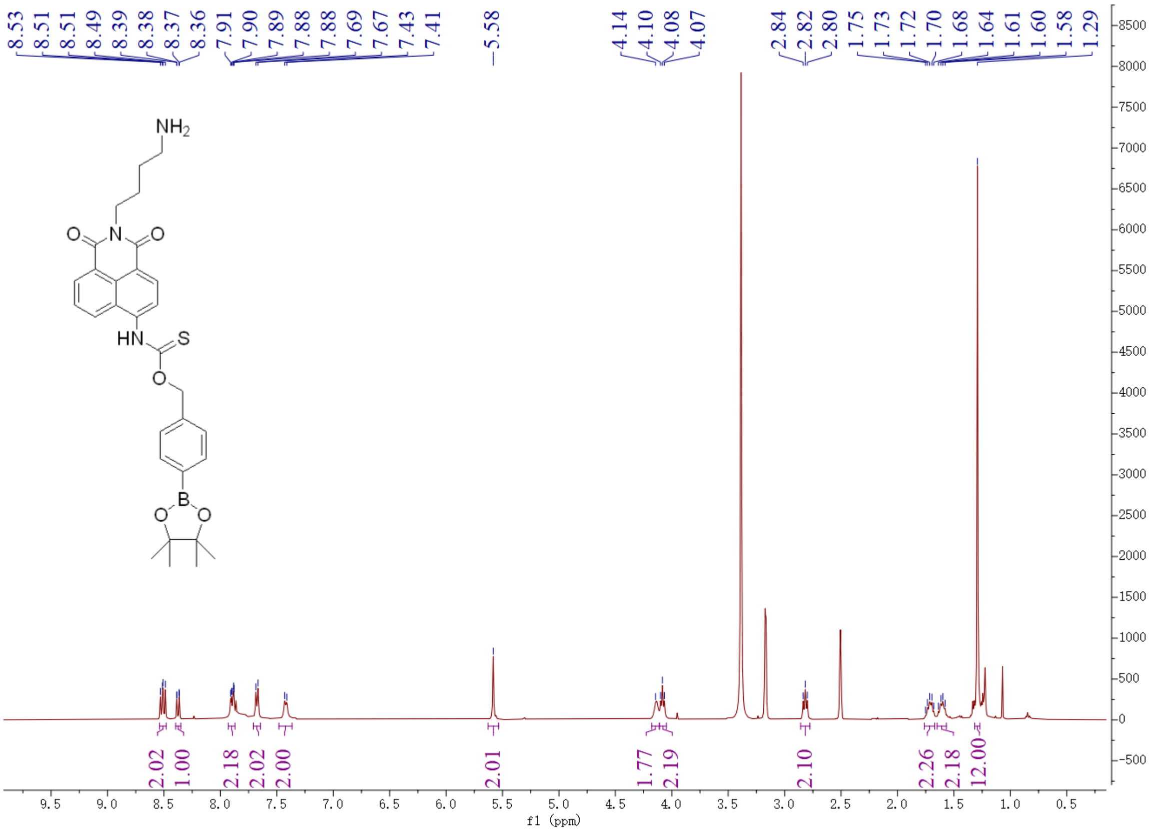

Figure S20. ^{1}H NMR spectrum (400 MHz, DMSO-d_6) of HSDF-NH_2.

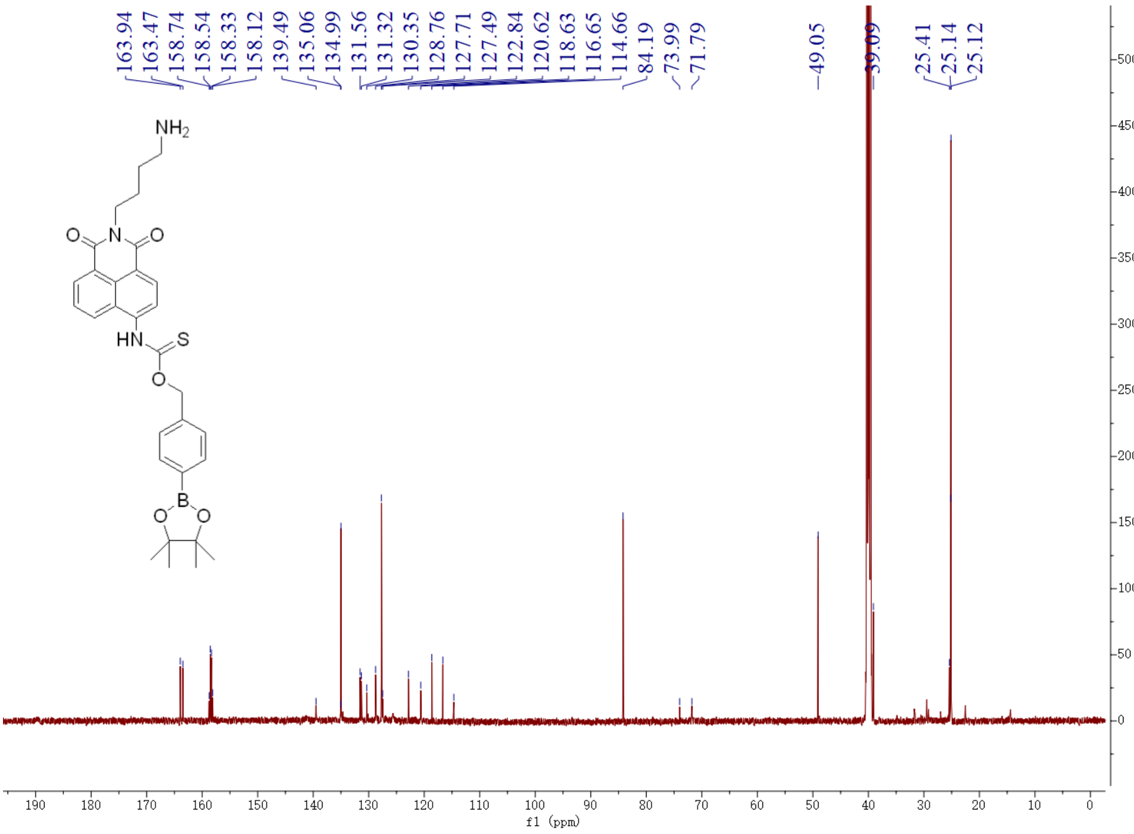

Figure S21. ^{13}C NMR spectrum (151 MHz, DMSO-d_6) of HSDF-NH_2.

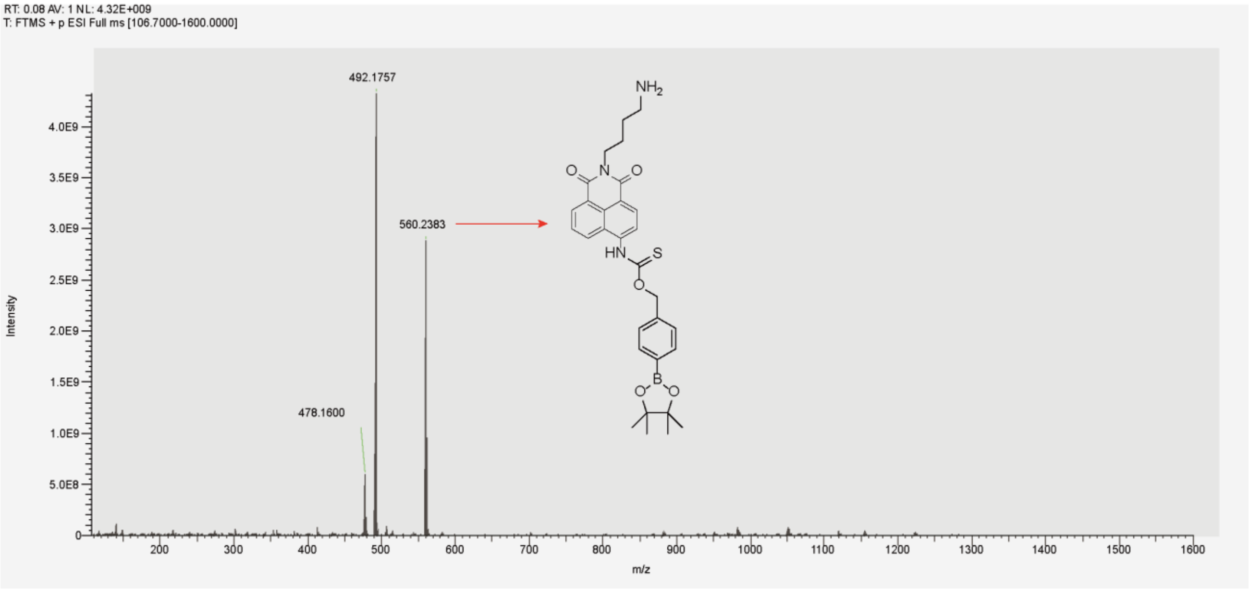

214

215 Figure S22. High-resolution mass spectrum of HSDF-NH_2.

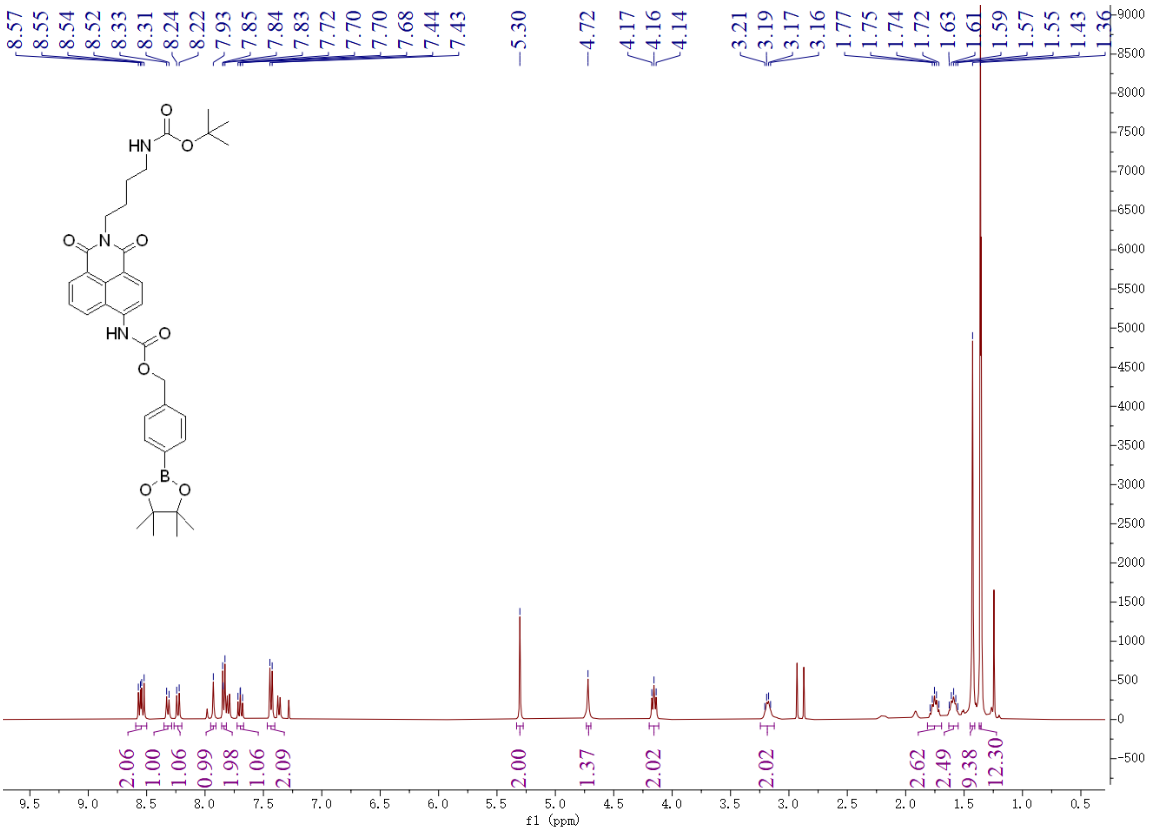

Figure S23. ^{1}H NMR spectrum (400 MHz, Chloroform-d) of compound **7**.

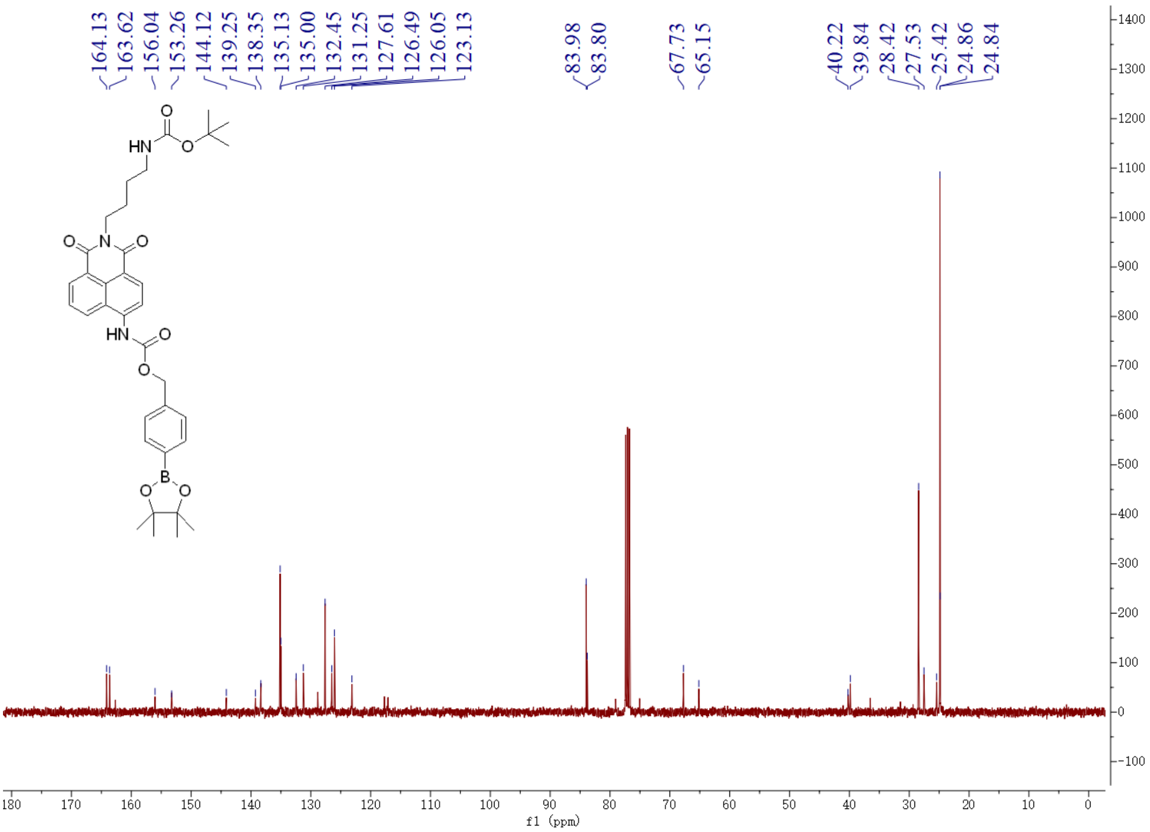

Figure S24. ^{13}C NMR spectrum (101 MHz, Chloroform-d) of compound **7**.

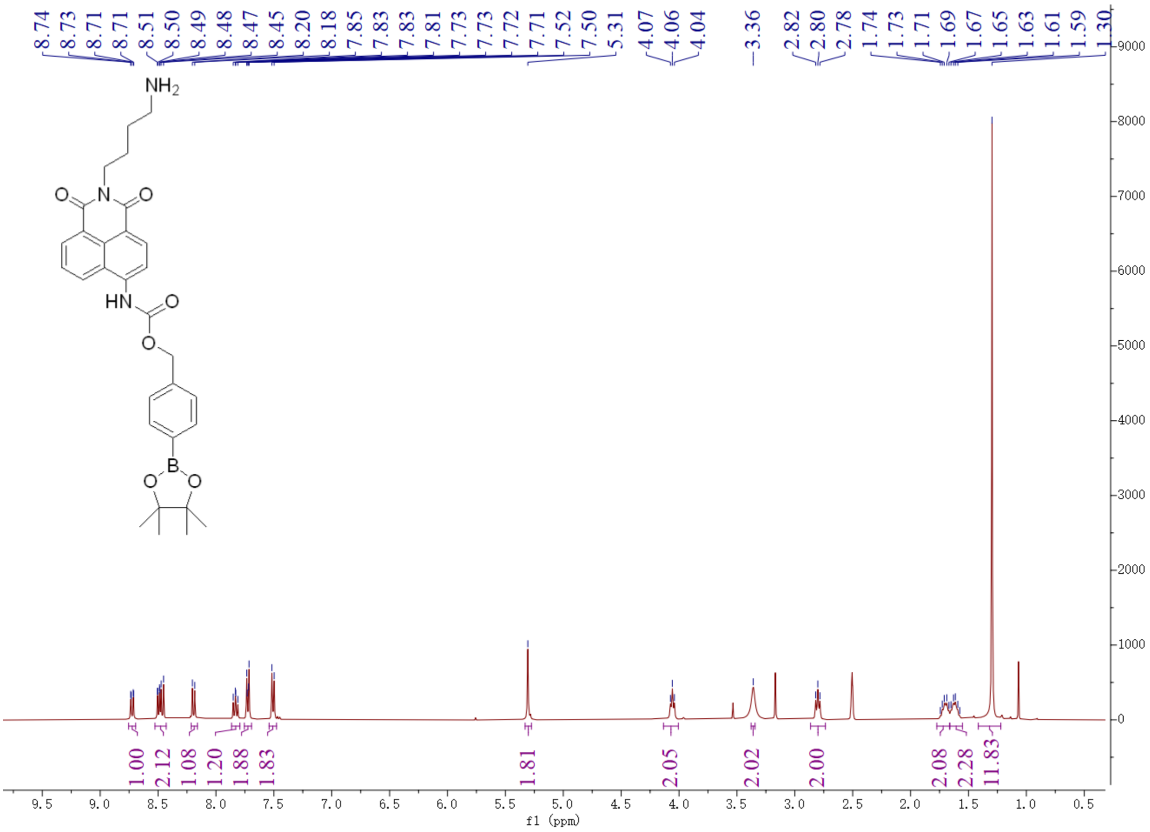

Figure S25. ^{1}H NMR spectrum (400 MHz, DMSO-d_6) of CODF-NH_2.

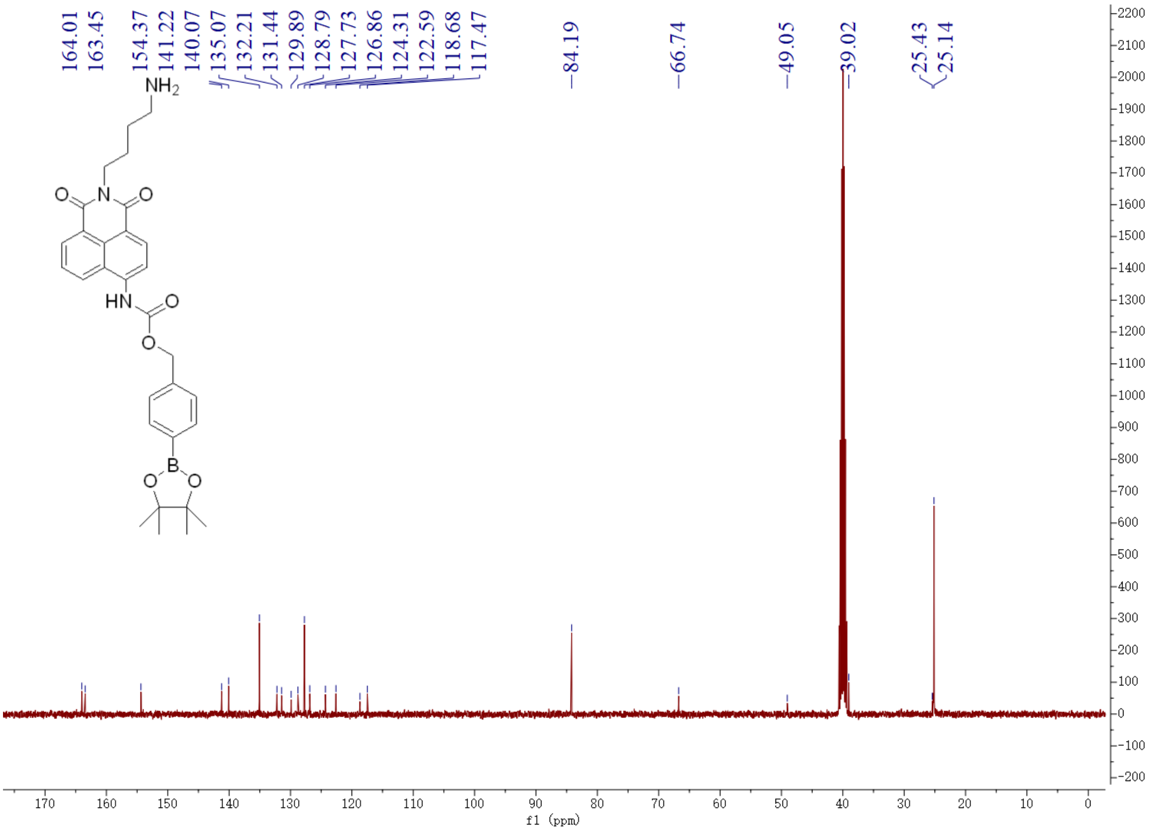

Figure S26. ^{13}C NMR spectrum (101 MHz, DMSO-d_6) of CODF-NH_2.

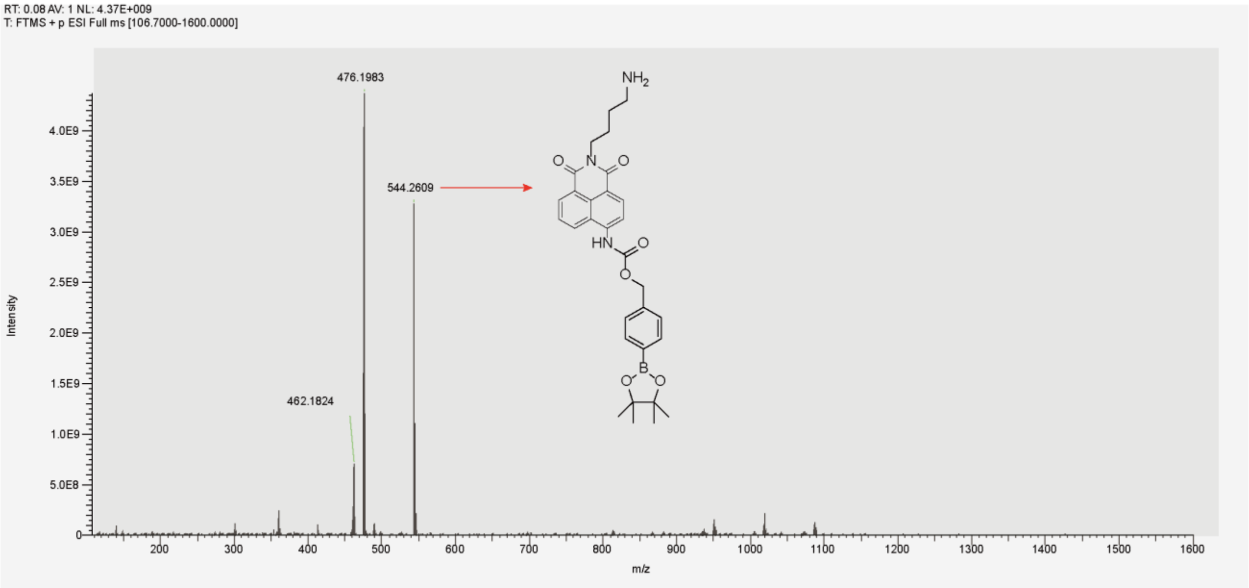

Figure S27. High-resolution mass spectrum of CODF-NH_2.

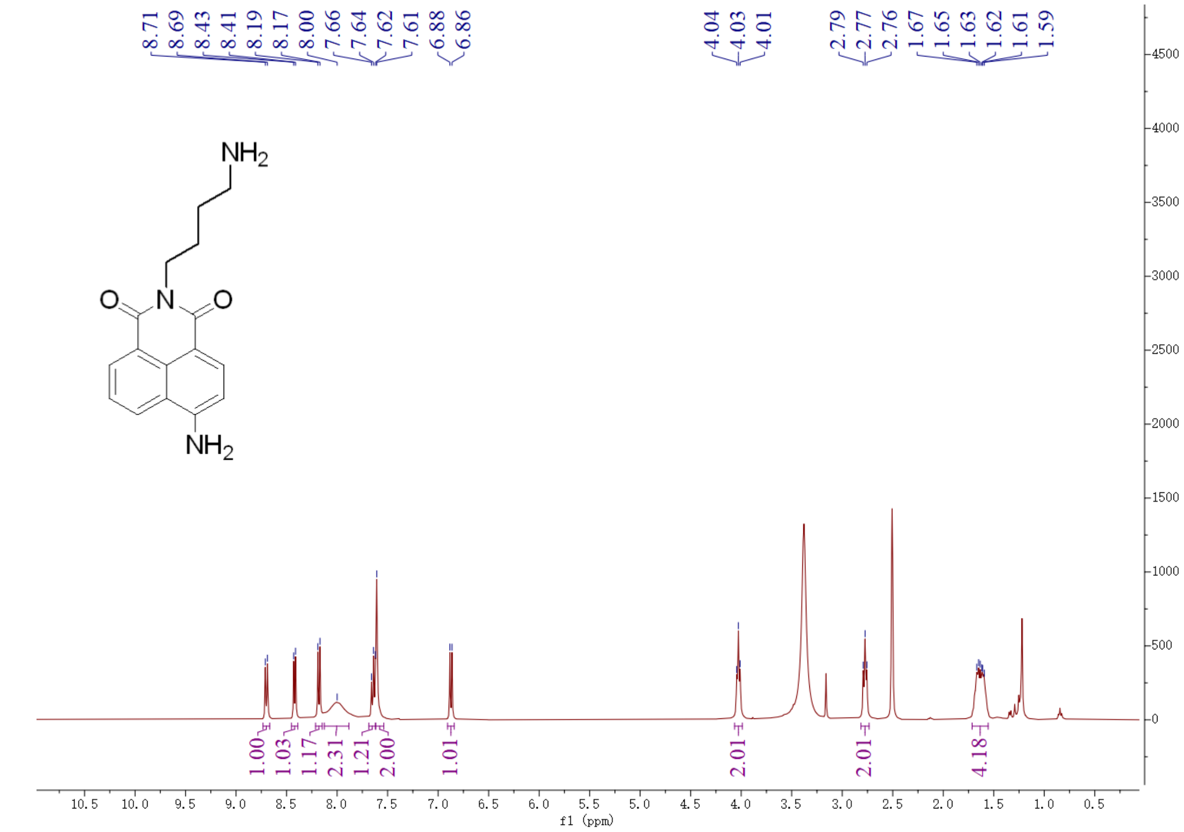

Figure S28. ^{1}H NMR spectrum (400 MHz, DMSO-d_6) of HSDG-NH_2.

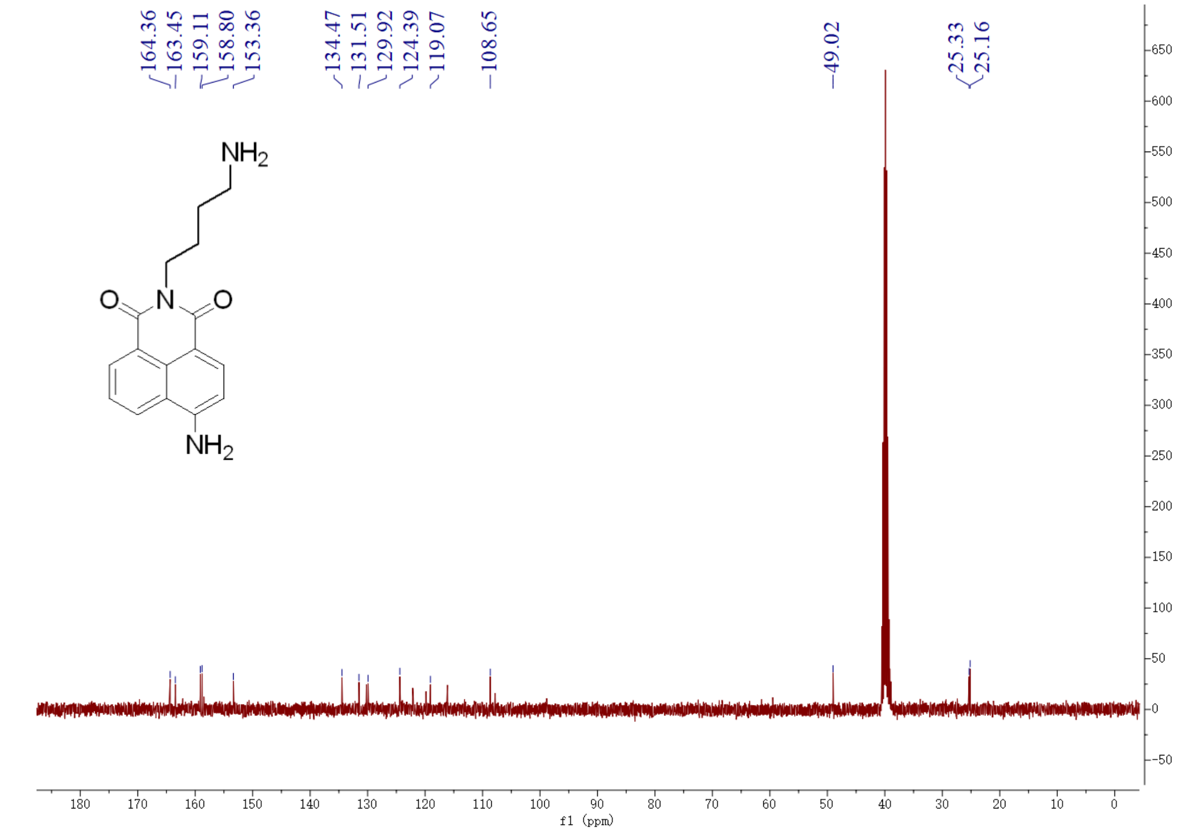

Figure S29. ^{13}C NMR spectrum (101 MHz, DMSO-d_6) of HSDG-NH_2.

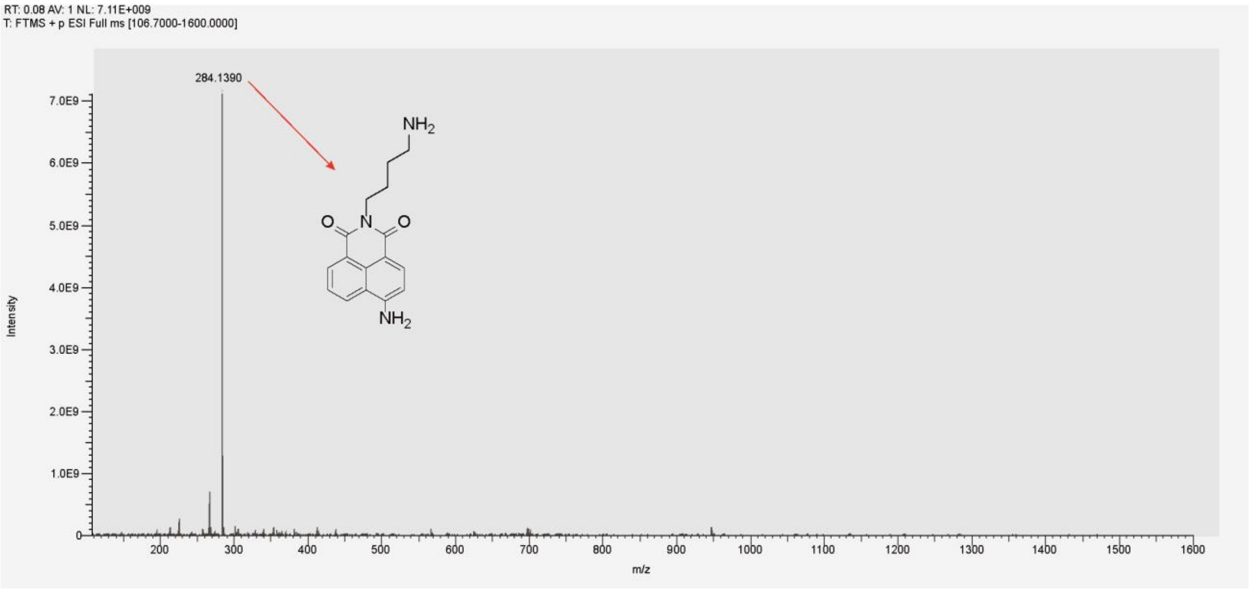

Figure S30. High-resolution mass spectrum of HSDG-NH_2.

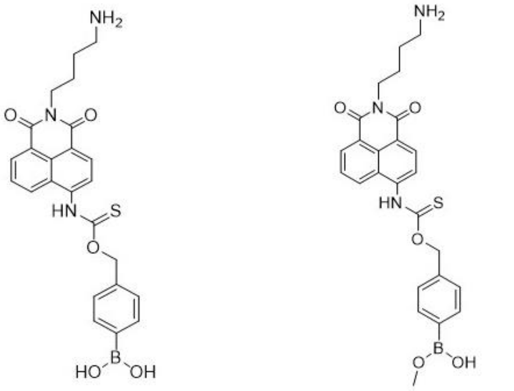

M/Z: 478 M/Z: 492

Figure S31. Possible structures of the ionized fragments of HSDG-NH_2 in mass spectrometry.

S6 Data supplementation

Table S1. Precision of the data (A：model/ OGD/R　B：CONF-NH_2　C：HSDF-NH_2)

Figure 3G	A	B	C	P	
	42.13	37.08	18.75	A vs B	0.3423
	43.91	41.36	20.82	A vs C	0.0001
	51.01	44.92	26.22		
Figure 3H	A	B	C	P	
	3	2	1	A vs B	0.3802
	2	3	2	A vs C	0.0017
	3	2	1		
	3	3	2		
	3	2	2		
Figure 5F	A	B	C	P	
	24	22	12	A vs B	0.4943
	11	23	18	A vs C	0.0074
	31	26	10		
	25	18	14		
	37	18	13		
Figure 5G	A	B	C	P	
	51	42	34	A vs B	0.1469
	47	41	27	A vs C	< 0.0001
	57	54	32		
	59	50	35		
	45	40	37		
Figure 5H	A	B	C	P	
	23.67	18.68	11.28	A vs B	0.5248
	25.21	29.06	10.66	A vs C	0.0055
	32.00	31.08	15.00		
	29.00	29.67	13.83		
	25.00	3.82	13.04		
Figure 5I	A	B	C	P	
	0.206	0.244	0.124	A vs B	0.1594
	0.296	0.183	0.141	A vs C	< 0.0001
	0.291	0.267	0.145		
	0.335	0.183	0.163		
	0.253	0.277	0.091		
Figure S6	A	B	C	P	
	105.065	67.986	14.443	A vs B	0.0110
	115.969	77.131	17.478	A vs C	< 0.0001
	89.466	82.692	19.317		
